# Differences in the microbiota of oral rinse, lesion, and normal site samples from patients with mucosal abnormalities on the tongue

**DOI:** 10.1038/s41598-022-21031-8

**Published:** 2022-10-07

**Authors:** Yawaka Shitozawa, Kaoru Haro, Midori Ogawa, Akihiko Miyawaki, Mitsumasa Saito, Kazumasa Fukuda

**Affiliations:** 1 Department of Microbiology, School of Medicine, University of Occupational and Environmental Health, 1-1 Iseigaoka, Yahatanishi-ku, Kitakyushu, Fukuoka 807-8555 Japan; 2 Department of Dentistry and Oral Surgery, Hospital of the University of Occupational and Environmental Health, 1-1 Iseigaoka, Yahatanishi-ku, Kitakyushu, Fukuoka 807-8556 Japan

**Keywords:** Microbiology, Oral diseases

## Abstract

The oral microbiota associated with mucosal diseases, including oral squamous cell carcinoma and oral potentially malignant disorders, have been extensively analyzed at the phylum and genus levels. However, the details of the oral microbiota remain unclear at the species and operational taxonomic unit (OTU) levels. We aimed to determine differences in the microbiota of oral rinse, lesion and normal site swab samples of patients with mucosal abnormalities on the tongues. Oral samples were obtained from 10 patients with oral mucosal abnormalities. Alpha and beta diversity at the OTU and genus levels of the microbiota samples were analyzed using OTUs clustered with 99.6% similarity based on 16S rRNA gene sequences obtained using the Sanger method. At the OTU level, the microbiota of the lesions were the least diverse but were different from those of the normal site and oral rinse samples. The OTUs corresponding to *Streptococcus infantis* and *Haemophilus parainfluenzae* were suggested to contribute to the differences between the microbiota of the lesions and normal sites. At the genus level, no significant differences between these microbiota were observed. In conclusion, strict OTU-level microbiota analysis might be able to discriminate lesions from normal sites of patients with mucosal abnormalities.

## Introduction

In 2020, 377,713 new patients with oral cancer were reported worldwide, of which 177,757 died^1^. In 2018, 22,515 Japanese individuals were diagnosed with oral cavity and pharynx cancer, and 7827 Japanese patients died as a result of these conditions in 2020 (https://ganjoho.jp/reg_stat/statistics/data/dl/en.html). Oral squamous cell carcinoma (OSCC) accounts for more than 90% of oral cancers^2–4^. In addition to smoking and drinking, oral bacteria have been reported to be associated with oral cancer^2,5,6^. The involvement of oral bacteria has also attracted attention in terms of malignant changes from oral potentially malignant disorders (OPMD) to OSCC^7,8^.

Human microbiota in both health and disease have been analyzed using next-generation DNA sequencing (NGS)^9^. The oral microbiota in many systemic and oral diseases have also been investigated using NGS^10^. Analysis of the oral microbiota associated with oral mucosal disease has been extensively reported. In these studies, saliva^11–13^, oral rinse sample/mouthwash^14–16^, oral cancer tissues^17–20^, swabs^21–24^ and various clinical specimens^25^ were used as the target specimens. The 16S rRNA gene was used as the target gene in most of these studies, but the primer sets used for PCR differed^11,15,18,20,21,25^. Using NGS, nucleotide sequences after filtering and trimming were generally clustered into operational taxonomic units (OTUs) with 97 or 98% similarity and applied to microbiota analysis at the phylum and genus levels. Generally, oral bacteria include a genus comprising many closely related species, such as *Streptococcus*; however, the details of the species levels of the oral microbiota in oral mucosal disease have not been fully elucidated. Segata et al. reported that the microbiota in buccal mucosa, keratinized gingiva, and hard palate are different from those of the dorsal tongue and saliva^26^. Although microbiota in buccal mucosal cancer^23^ and gingival squamous cell carcinoma^25^ have been analyzed using NGS, details of the microbiota of the tongue have not been fully analyzed, yet the tongue is the most common site for oral cancer^27^.

In this study, to clarify the details of the oral microbiota associated with oral mucosal diseases, we obtained swab and oral rinse samples from patients with oral mucosal abnormalities on the tongue and analyzed the microbiota in the samples using highly accurate 16S rRNA gene sequencing based on Sanger’s method.

## Results

### Subject characteristics

The characteristics and clinical diagnoses of the 10 patients are shown in Table 1 and Supplementary Fig. S1. Thirty samples were analyzed in total. The median age of the patients (7 men and 3 women) was 68 years (range 50–92 years). The patients included two diagnosed with OSCC and eight diagnosed with OPMD according to the World Health Organization classification.

**Table 1:** Characteristics of the patient population.

Patient no.	Gender (M/F)	Age	Clinical diagnosis (OSCC/OPMD)	Pathological diagnosis	Site of lesion on the lateral border of the tongue
1	M	85	OPMD	–	Left
2	M	60	OSCC	Squamous cell carcinoma	Right
3	F	92	OPMD	Mild dysplasia	Right
4	M	65	OPMD	Mild dysplasia	Right
5	F	55	OSCC	Squamous cell carcinoma	Right
6	M	77	OPMD	Severe dysplasia	Left
7	F	71	OPMD	Moderate dysplasia	Left
8	M	60	OPMD	Severe dysplasia	Left
9	M	87	OPMD	Mild dysplasia	Right
10	M	50	OPMD	Severe dysplasia	Left

*M* male, *F* female, *OSCC* oral squamous cell carcinoma, *OPMD* oral potentially malignant disorders.

^a^Patient no. 1 Subject did not wish to have a histological examination.

### Total bacterial cell counts

The total bacterial cell counts are shown in Supplementary Fig. S2. The average numbers of bacteria in the swab samples were 1.7 × 10^7^ cells/ml (ranging from 1.5 × 10^6^ to 3.2 × 10^7^ cells/ml) in the lesions and 8.3 × 10^6^ cells/ml (ranging from 5.2 × 10^5^ to 2.2 × 10^7^ cells/ml) in the normal sites (Supplementary Fig. S2a). No significant difference was observed between the bacterial cell counts of the swabs of the lesions and the normal sites (p > 0.05) (Supplementary Fig. S2b). The average number of bacteria in the oral rinse samples was 1.0 × 10^8^ cells/ml (ranging from 3.5 × 10^7^ to 2.2 × 10^8^ cells/ml) (Supplementary Fig. S2c).

### OTU level analysis

**Figure 1:**
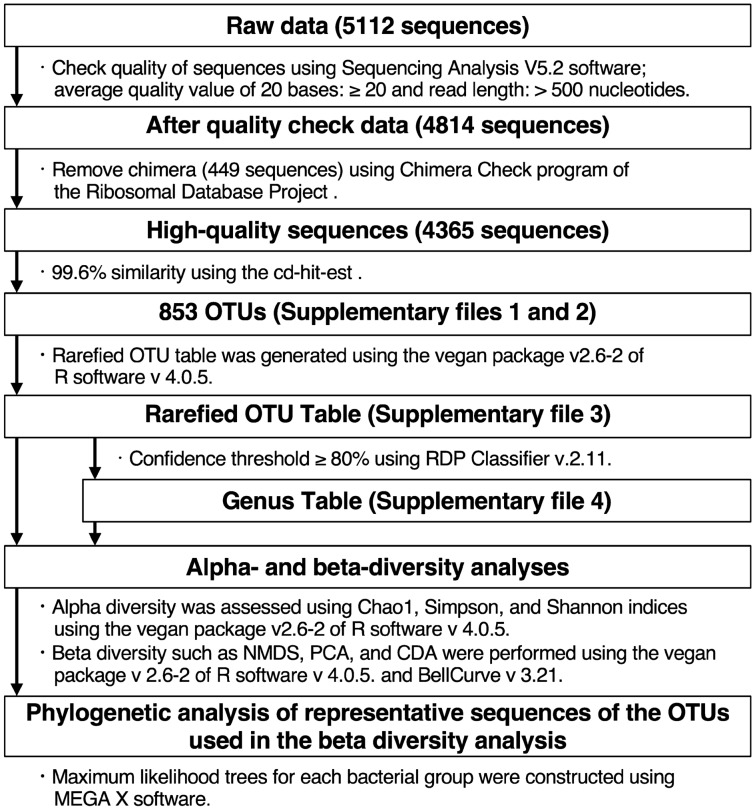
Summary of data analysis. *OTU* operational taxonomic unit.

**Figure 2:**
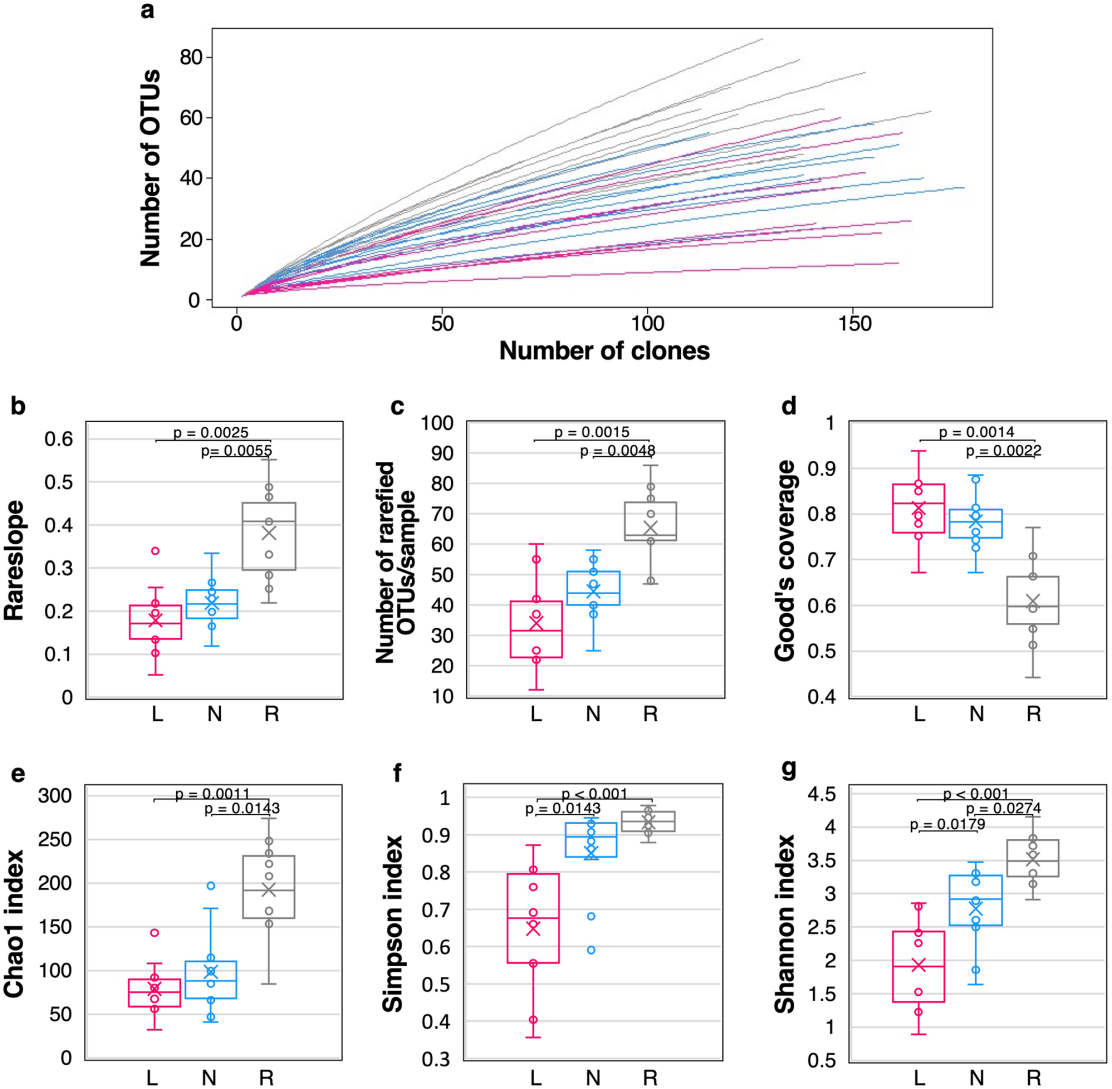
Rarefaction analyses and comparison of alpha diversity of OTU level. (**a**) Rarefaction curve. Lesion samples are drawn as pink lines, normal-site samples as blue lines, and oral rinse samples as gray lines. (**b**) Rareslope, (**c**) number of rarefied OTUs/sample, (**d**) Good’s coverage, (**e**) Chao1 index, (**f**) Simpson index, and (**g**) Shannon index. (**b–g**) The box plots show the 25th and 75th percentiles (bottom and top of the box, respectively), median (middle horizontal line), average (cross mark) and minimum and maximum values that are not outliers (top and bottom whiskers). Outliers were defined as values greater than 1.5× the interquartile range (points). Statistical significance among the three sample types was assessed using the Kruskal–Wallis test. For multiple comparisons, the Steel–Dwass test was used (bars, p < 0.05). *L* lesion, *N* normal site, *R* oral rinse, *OTU* operational taxonomic unit.

In this study, 4814 highly accurate sequence reads were obtained from 30 samples (Fig. 1). After removing chimeric sequences (449 sequences), the remaining 4365 sequence reads were clustered into 853 OTUs with 99.6% similarity (Supplementary Files S1, S2). The rarefaction curves of the swab samples showed near saturation more than that of the oral rinse samples (Fig. 2a). The relative abundance of OTUs based on rarefied OTU table in all samples showed that the OTU composition was quite different in individual patients and sample types (Supplementary File S3, Supplementary Fig. S3). The rareslope values of the swab samples were also significantly lower (p < 0.05) than those of the oral rinse samples (Fig. 2b). The average numbers of OTUs detected in the lesions, normal sites, and oral rinse samples were 34.0 (ranging from 12 to 60 OTUs), 44.5 (ranging from 25 to 58 OTUs), and 65.4 (ranging from 47 to 86 OTUs), respectively (Fig. 2c). Good’s coverage estimates for lesions and normal sites averaged 0.81 (ranging from 0.67 to 0.94) and 0.78 (ranging from 0.67 to 0.88) (Fig. 2d), respectively. The number of OTUs was lower in the lesion and normal site swab samples than in the oral rinse ones (p < 0.05), while Good’s coverage estimation values were significantly higher in the lesion and normal site swab samples than in the oral rinse ones (p < 0.05).

The results of the α-diversity evaluation using OTUs are shown in Fig. 2e–g. Among the three indices, Chao1, Simpson and Shannon, the α-diversity of the oral rinse samples was significantly higher than that of the lesion and normal site samples (p < 0.05). Moreover, both the Simpson and Shannon indices were lower in the lesion swabs than in the normal site swabs (p < 0.05). These results clearly show that the microbiota of the lesion swab were less diverse than those of the normal site swab and oral rinse samples.

**Figure 3:**
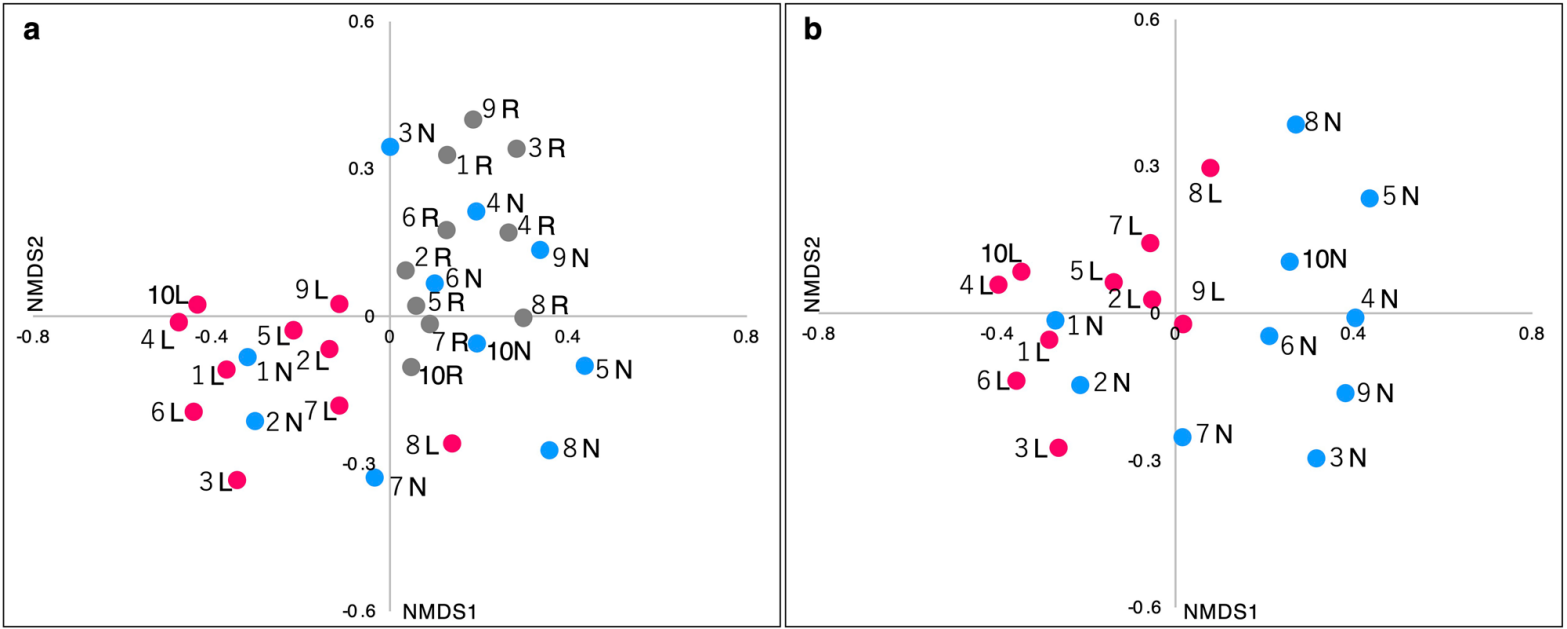
Nonmetric multidimensional scaling plots based on OTU level. (**a**) Three sample types. (**b**) Two swab samples. Nonmetric multidimensional scaling plots were generated from a distance matrix of Bray–Curtis dissimilarity values based on OTU level. Each point corresponds to a single sample. Lesion samples are plotted as pink dots, normal-site samples as blue dots, and oral rinse samples as gray dots. The data labels show the patient number. *L* lesion, *N* normal site, *R* oral rinse, *OTU* operational taxonomic unit.

**Figure 4:**
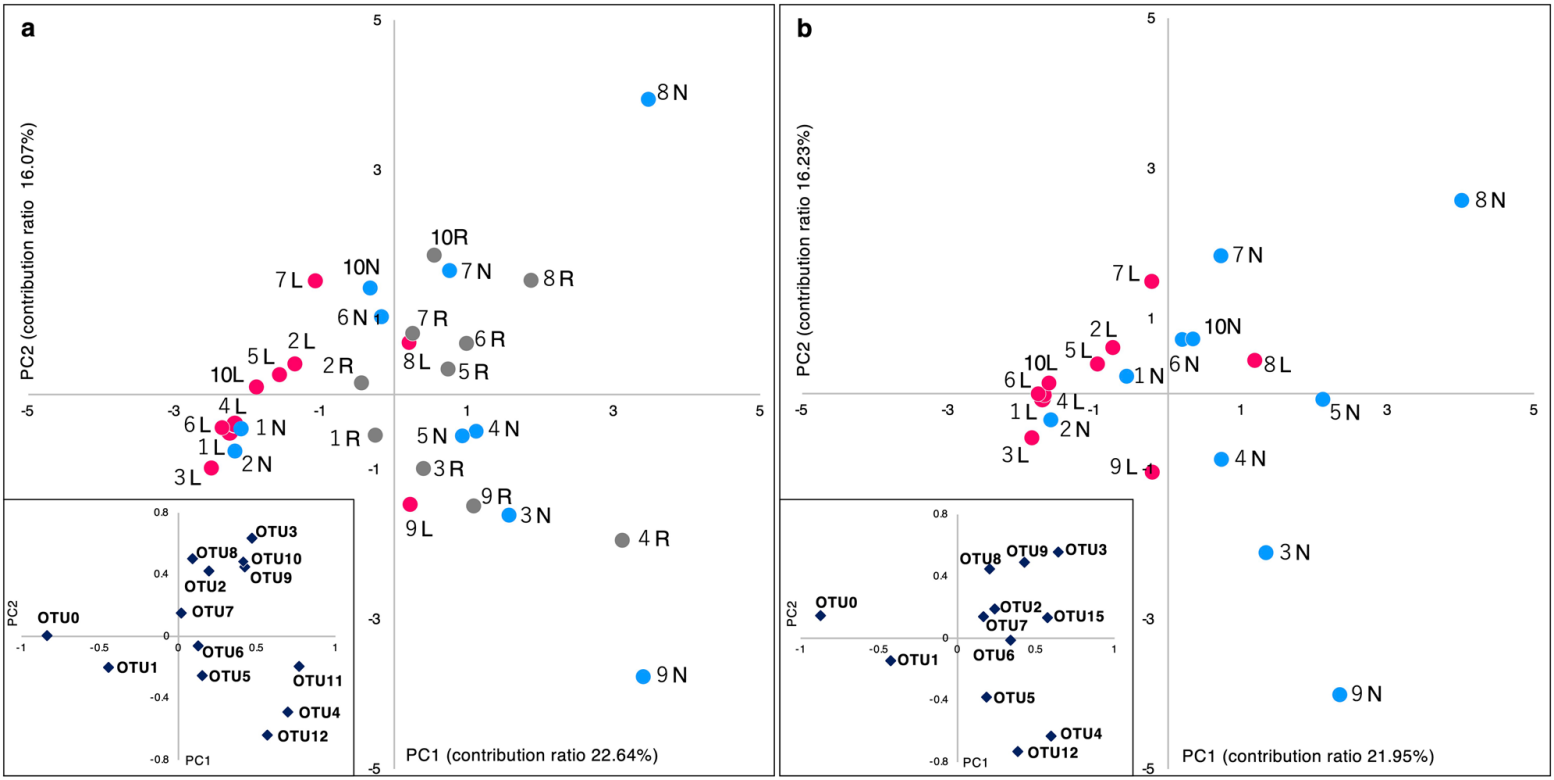
Principal component analysis based on OTU levels. (**a**) Three sample types. (**b**) Two swab samples. The horizontal axis shows PC1, and the vertical axis shows PC2. The eigenvector is shown in the box below. Others are OTUs with relative abundance < 1% in (**a**) the total sequences from all samples and (**b**) the total sequences from lesions and normal sites. Each point corresponds to a single sample. Lesion samples are plotted as pink dots, normal-site samples as blue dots, and oral rinse samples as gray dots. The data labels show the patient number. *L* lesion, *N* normal site, *R* oral rinse, *OTU* operational taxonomic unit, *PC1* first principal component, *PC2* second principal component.

The β-diversity based on the OTUs of all samples was evaluated using three multivariate analyses. The nonmetric multidimensional scaling (NMDS) analysis using the Bray–Curtis dissimilarity index showed a tendency for the spots of the lesion swabs to cluster independently from the normal-site swabs and the oral rinse samples (Fig. 3a,b). In the principal component analysis (PCA) results (Fig. 4), the spots of the normal site and the oral rinse samples were distributed in the positive direction of the first principal component (PC1) (Fig. 4a). In contrast, the spots of the lesion swabs were scattered in the negative direction of the PC1. Since one of the representative eigenvectors had a large effect in the negative direction of the PC1 at OTU 0, the bacterium corresponding to it might be a key factor in distinguishing the microbiota of the lesions. Comparing only the swab samples (Fig. 4b), the spots of the lesions and normal sites were also clearly separated, and the strong effect of OTU 0 as an eigenvector in the distinction is suggested.

**Figure 5:**
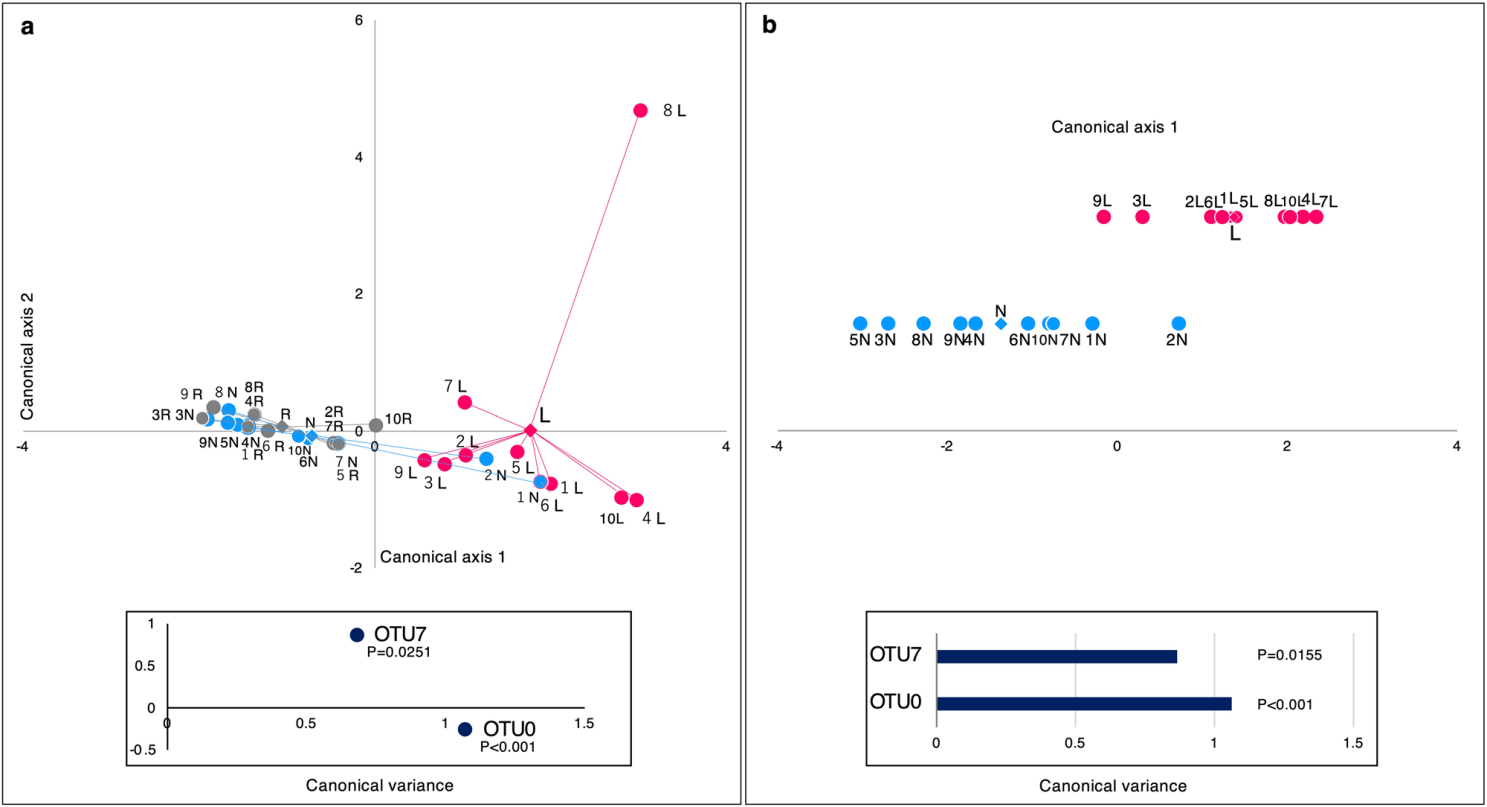
Canonical discriminant analysis based on rarefied OTU level. (**a**) Three sample types. (**b**) Two swab samples. Each point represents the canonical score (CS) of each sample based on the respective canonical variables. The X-axis is canonical axis 1, and the Y-axis is canonical axis 2. Each point corresponds to a single sample. Lesions are plotted as pink dots, normal sites as blue dots, oral rinse samples as gray dots, and each center of gravity as diamonds. Data labels show the patient number. *L* lesion, *N* normal site, *R* oral rinse. Others are OTUs with relative abundance < 1% in (**a**) the total sequences from all samples and (**b**) the total sequences from lesions and normal sites. *OTU* operational taxonomic unit. Canonical discriminant function p < 0.05.

To detect the factors that are effective in discriminating lesion swabs from other samples, canonical discriminant analysis (CDA) was applied to these samples (Fig. 5). The effects of OTUs 0 and 7 as discriminant factors in distinguishing lesion swabs from other samples in the CDA are shown in Fig. 5a,b (p < 0.05). These results indicate that the lesion samples harbored different microbiota from the normal site and the oral rinse samples. Moreover, it was revealed that the bacterial species corresponding to OTUs 0 and 7 might contribute to the discrimination between the lesions and normal sites. After the estimation using the classifier on the RDPII website for the genera of the representative sequences of OTUs 0 to 12, and 15 (Supplementary Table S1), phylogenetic analyses based on a 16S rRNA gene sequence containing type strains of each genus were performed (Supplementary Figs. S4–S10). ML trees showed that OTUs 0 and 7 were phylogenetically close to *Streptococcus infantis* and *Haemophilus parainfluenzae,* respectively.

### Genus level analysis

At genus-level analyses, the three genera: *Streptococcus* (57.52%), *Gemella* (8.76%), and *Neisseria* (6.37%) accounted for 72.65% of the total sequences (Supplementary File S4, Supplementary Fig. S11). Whereas, the genera *Fusobacterium* (2.98%) and *Prevotella* (2.45%), which include several known periodontal pathogens, accounted for less than 3% of the total sequences in this study. The α-diversity analyses at the genus level showed that the lesion swabs were significantly lower than that of the oral rinse samples on the Chao1 index (p < 0.05) (Supplementary Fig. S12). In the results of NMDS and PCA based on the genus level, no significant differences were observed among these samples (Supplementary Figs. S13, S14). The results of the CDA showed the contribution of genera *Neisseria, Veillonella,* and *Sphingomonas* to the discrimination between the three sample types (p < 0.05) (Supplementary Fig. S15a). The genus *Veillonella* was designated as one of the factors to distinguish the normal microbiota from lesions (p < 0.05) (Supplementary Fig. S15b). However, no factors specific to lesion samples were identified.

## Discussion

This is the first study to analyze the microbiota of visible oral mucosal abnormalities of the tongue, including OSCC and OPMD, using high-accurate 16S rRNA gene sequences based on Sanger sequencing and strict OTU clustering with nearly 100% similarity. It reveals that the microbiota in swab samples of lesions of the tongue, normal sites of the tongue, and oral rinse samples from patients with visible oral mucosal abnormalities were significantly different.

Many studies based on NGS regarding the microbiota in the oral cavity have been conducted using saliva or oral rinse samples^7,12,14,15^. Based on the OTU levels in this study, the microbiota of the oral rinse samples were different from that of the swab samples of lesions. This suggests that the microbiota of oral rinse samples are not representative of the lesions, and oral rinse samples are not suitable for investigating the factors of the microbiota of lesions. Previous studies have suggested the association of OSCC and OPMD lesion sites with *Fusobacterium*^18,21–24^, *Alloprevotella*^23^, *Porphyromonas*^18,23^, *Prevotella*^17,20^, *Corynebacterium*^17^, *Pseudomonas*^17,18^, and *Peptostreptococcus*^22^, and normal sites with *Streptococcus*^18,20–23^ and *Veillonella*^20^. Most studies have analyzed at the genus and OTU levels (almost 97 or 98% similarity), but the results and conclusions were different in each study. Thus, bacterial factors that predict OSCC and OPMC have not been fully elucidated.

In this study, the genus *Streptococcus* was the most predominant in all three sample types. This was consistent with the results of studies of saliva in healthy subjects^28^, saliva and oral rinse samples in OSCC patients^15,16^, and swabs in OSCC patients^23^. It is well known that there are many different bacterial species belonging to the genus *Streptococcus* with very similar 16S rRNA gene sequences in the oral cavity. Identifying these bacterial species and analyzing microbiota in detail are not possible with OTUs that cluster with 97 and 99% similarity^29^. In most of previous studies using NGS technology, the OTUs clustered with 97 or 98% similarity were used, and the analysis was limited to the genus level. The V3–V5 region is better than the V3–V4 region for the identification of bacteria at the species level, one example being the *Streptococcus* species^30^. Since the Sanger method can read comparatively long sequences and is more accurate per read than other NGS methods^31^, this method is currently suitable for obtaining a high-precision base sequence of the V3–V5 region of approximately 600 bp. To understand the correlation between mucosal diseases, such as OSCC and OPMD, and the bacterial communities, it is essential to elucidate the microbiota in the oral cavity based on strict OTUs using highly accurate 16S rRNA gene sequences.

In a previous study, we performed OTU analysis of the highly accurate nucleotide sequence, as determined by the Sanger method, with 99.6% similarity, and demonstrated its ability to discriminate the microbiota in nasal discharge at the species or strain level^32^. This analysis protocol was applied to the oral microbiota study, resulting in less α-diversity in the lesion microbiota compared to the normal sites, and oral rinse samples were revealed. This outcome is similar to the results of previous studies based on NGS^12,17^, but different from that of Zhao et al.^22^. Of particular interest in this study was that the use of strict OTUs in β-diversity analyses (NMDS, PCA and CDA) could discriminate between the lesions and normal sites, and candidates for distinctive bacterial factors were identified. An OTU corresponding to a species of the genus *Streptococcus*, presumed to be *S. infantis* (OTU 0), had a significant effect on lesion convergence in both PCA and CDA. A significant effect of OTU corresponding to *H. parainfluenzae* (OTU 7) in the lesions was also suggested. According to the results of previous NGS studies, the genera *Streptococcus* and *Haemophilus* were more common in normal sites than in lesions, which is inconsistent with the results of this study^18,20–23^. In healthy individuals, *S. infantis* was reported to demonstrate site-specific colonization of the tongue, but rarely of subgingival plaques^33^. In contrast, *S. infantis* has been suggested as a potential biomarker of gastritis, including precancerous pathology, by metagenomic analysis of tongue coating samples^34^. Further analysis is needed regarding the relationship between the lesions of mucosal diseases including OSCC and OPMD; however, *S. infantis* may be a predictor of these diseases. Although genus-level analysis is usually used in NGS analysis, the results at the genus level in this study did not clearly characterize the microbiota of the lesions and normal sites. The discrepancy between the results of this study and the other NGS studies is not only the difference in the sample types and in the variable region of the target 16S rRNA gene, but also the OTU clustering similarity. Therefore, the important factors involved in the convergence of the microbiota can be identified by analyzing the OTU sequences before bacterial classification of the obtained OTUs.

In some cases, such as those of patients 1 and 2, lesions and normal sites were exceptionally unclear distinctions in β-diversity. In these cases, the difference in the bacterial cell counts between the lesions and normal sites might have affected the results. It has been reported that bacterial cell numbers affect the onset of the disease^35^. Further investigation is needed to determine the relationship between the lesions and bacterial cell counts. Alternatively, it was possible that patients 1 and 2 might have been prone to developing multiple lesions in the oral cavity, although it could not be visually identified.

This study had some limitations. First, the area rubbed with a swab was 1 cm^2^, making it difficult to collect smaller lesion samples. Second, the number of clones analyzed in this study was up to 192 per sample, and this method may not be able to detect bacterial species in a minor population. In fact, rarefaction analysis showed that the sample size was not sufficient to cover the entire microbiota. However, since our main purpose was to assess the definite differences in the microbiota of the oral samples, this effect might not be remarkable. Third, due to the limited sample size of 10 patients, the microbiota of OSCC and OPMD could not be compared statistically. In this study, it was not possible to determine whether changes in the oral microbiota of the lesion caused cancer development or whether malignant transformation of the oral mucosa caused by other factors altered the microbiota of the lesion.

In conclusion, the analysis based on the OTU level revealed the characteristics of the microbiota of oral lesions; however, the analysis based on the genus level could not distinguish the microbiota of the lesions from those of the normal sites. These results clearly show that the investigation of bacterial lineage at a higher resolution than the genus level, such as species or OTU level, might be important to distinguish oral site samples.

## Methods

### Participant enrollment

A single-center prospective observational study was conducted. Patients aged 20 years and older with visible oral mucosal abnormalities on the lateral border of the tongue who visited the Department of Dentistry and Oral Surgery at the Hospital of the University of Occupational and Environmental Health, Japan from 2018 to 2020 were enrolled. Clinical information such as sex and age was collected at the time of enrollment. Samples were collected from patients prior to biopsy and antibiotic treatment.

### Sample collection

To eliminate sampling errors due to individual differences, swabs and oral rinse samples were collected by a single dentist. Swabs were collected from the mucosal surface with macroscopic lesions and from an anatomically opposite normal site on the tongue. A 1 cm square paper L-shaped caliper was used to collect a swab sample from a certain area (approximately 1 cm^2^) of the lesions and normal sites. The surface of the sampling site was dried, and a certain area per 1 cm^2^ was rubbed with a sterile cotton swab ten times and then stored in a 15 ml sterile tube. Then, the oral rinse sample, which was rinsed with 5 ml of sterilized distilled water for 10 s, was collected in a 50 ml sterile tube. All samples were immediately stored at 4 °C until the next procedure (within 1 d). Before the next procedure, to prepare a suspension of bacterial cells on a swab, 1 ml phosphate buffered saline was added to a swab in a 15 ml tube and vortexed for 15 s. Subsequently, it was ultrasonically treated using a Branson ultrasonic washing machine (Branson Ultrasonic Corporation) for 5 min.

### Ethics statement

This study was approved by the Ethics Committee of Medical Research of the University of Occupational and Environmental Health in Japan (UOEHCRB21-119). All methods in this study were performed in accordance with relevant guidelines and regulations. Informed written consent was obtained from all patients.

### Epifluorescence staining

All bacterial cells in the sample were counted using the epifluorescence staining method, as described previously^36^. An aliquot of the bacterial suspension (50 μl) from swab sample or oral rinse sample (10 μl) was filled up to 1000 μl with a buffer (0.1 M phosphate buffer [pH 8.5], 5% NaCl, 0.5 mM sodium EDTA). Then, 50 μl (2.0 mg/ml) of an ethidium bromide (EtBr) solution was added to 1 ml of the solution. After staining for 20 min, the bacterial cells were filtered through a 0.2 μm pore size filter and washed with 2 ml of sterile phosphate buffered saline (PBS). Microscopic examination was performed using the Olympus BX40 epi-illumination fluorescence microscope (Olympus, Tokyo, Japan), and the number of bacteria in 30 fields on the filter was counted. The counts of bacteria per 1 ml of bacterial solution prepared from the swab and oral rinse samples were calculated.

### DNA extraction

A 640 μl aliquot of the bacterial cell suspension from the swab and a 400 μl aliquot of the oral rinse sample were filled up to 1440 μl with PBS, and then 160 μl of a 30% SDS (final concentration, 3.0%) solution was added, respectively. After treatment with an Astrason model XL2020 (MISONIX Inc., Farmingdale, NY, USA) once for 15 s and three times for a total of 45 s (20 kHz), a 700 μl aliquot was mixed with 400 μl of TE-saturated phenol (Nacalai Tesque, Kyoto, Japan) and centrifuged at 15,000 rpm for 5 min. The extracted DNA in the supernatant was washed twice with PBS and TE buffer and concentrated (final volume of approximately 40 μl in TE buffer) using an Amicon Ultra-100 K filter (Merck Millipore Ltd., USA). DNA extracted from 640 μl of PBS using the same procedures described above was used as a negative control template.

### Polymerase chain reaction

The partial 16S rRNA genes (approximately 580 bp including V3-V5 variable regions) were amplified using the universal primer set E341F and E907R^37^, and AmpliTaq Gold DNA Polymerase LD (Applied Biosystems, USA), as described previously^36^. A single DNA band from the template DNA extracted from specimens and no DNA band from the negative control template were confirmed using 2% agarose gel electrophoresis.

### Clone library construction and nucleotide sequencing analysis

After confirming the PCR product, clone libraries were constructed using the TOPO TA cloning kit (Invitrogen, USA) according to the manufacturer’s instructions. The 192 colonies randomly selected from each sample were transferred into the wells of a PCR plate, and sequencing analysis was performed as described previously^36^. The inserts in the cloning vector (pCR 4 TOPO) were amplified using AmpliTaq Gold 360 DNA Polymerase (Applied Biosystems, USA) and a primer set (M13Forward: 5ʹ-GTAAAACGACGGCCAG-3ʹ and M13Reverse: 5ʹ-CAGGAAACAGCTATGAC-3ʹ). After removing unreacted primers and deoxyribonucleotide triphosphate using ExoSAP-IT (GE Healthcare UK Ltd., England, UK), the PCR mixture (1 μl) was used for the sequencing reaction using the BigDye Terminator Cycle Sequencing Kit v3.1 (Applied Biosystems). The nucleotide sequence was determined using the 3130xl Genetic Analyzer (Applied Biosystems). The quality of the sequence reads was evaluated using the Sequencing Analysis V5.2 software (Applied Biosystems)^38^. After the quality check (average quality value of 20 bases: ≥ 20 and read length: > 500 nucleotides), the sequences of the primers and vectors were trimmed from the remaining sequences. In addition, sequences containing ambiguous bases were excluded. The remaining highly accurate sequences (approximately 550 bp) were screened for chimeras using the Check Chimera program of the Ribosomal Database Project (RDP)^39^. All sequences that passed these examinations were registered in the DNA Data Bank of Japan (Accession Number: LC672676–LC677040). Then, the highly accurate sequences were clustered into OTUs^40^ using the cd-hit-est^41^ with 99.6% sequence similarity used as the threshold. After the treatment, rarefied OTU table was generated using the vegan package v2.6-2 of R software v 4.0.5. The representative sequences of OTUs were used for the bacterial composition analyses described below.

For the genus level analysis, the representative sequence of each OTU was classified using RDP Classifier v.2.11 at the genus level with a threshold of 80%^42^. Sequences with less than 80% at the genus level were classified as “unclassified bacterium” in the next highest taxonomic rank. The OTUs and genus that constituted less than 1% of the total number of sequences were grouped as ‘others’, respectively (Supplementary Tables S2, S3, S4, S5).

### Data analysis

All data analyses for this study were performed using R software v 4.0.5 and BellCurve v 3.21. Rarefaction curves based on OTUs were generated using the vegan package v2.6-2. The number of OTUs and Good’s coverage estimate^43^, based on the rarefied OTUs table, were calculated to evaluate the diversity coverage of each sample dataset. Alpha diversity based on OTU and genus levels was assessed using Chao1, Simpson, and Shannon indices using the vegan package v2.6-2. To evaluate the β-diversity among lesion swab samples, normal swab samples, and oral rinse samples, multivariate analyses such as NMDS, PCA, and CDA were performed using BellCurve v 3.21. NMDS was analyzed based on the distance matrix of the Bray–Curtis dissimilarity using the relative abundance of the OTU or the genus in each sample using the vegan package v 2.6-2. Similarly, PCA was performed using the relative abundance of the OTU or the genus in each sample. Moreover, a stepwise CDA was performed to identify significant OTUs or genus factors in lesion samples. The comparison between lesion and normal swabs was additionally demonstrated using multivariate analyses.

### Statistical analysis

Comparisons of bacterial cell counts between samples were performed using the Mann–Whitney *U* test. The Kruskal–Wallis test was used to evaluate the OTU number, Good’s coverage, rareslope, and α-diversity. The Steel–Dwass test was applied to compare the three types of samples. Statistical significance was set at p < 0.05. All statistical analyses in this study were performed using BellCurve software.

### Phylogenetic analysis

The bacterial lineages of the representative sequences of the OTUs used in the multivariate analyses were estimated using phylogenetic analysis. The closely related bacterial lineage to each sequence was estimated using the classifier software on the RDPII website, and the sequences of the type strains included in the genus were collected from the RDP database. Maximum likelihood trees for each bacterial group were constructed using MEGA X software. The sequences collected for each bacterial group (genus) were aligned multiple times using the MUSCLE algorithm. The best model for each phylogenetic analysis was selected using the Find Best DNA Models algorithm. The bootstrap support value for 500 bootstraps was displayed as a percentage for each section in the phylogenetic tree.

## Supplementary Information

Supplementary Information 1.

Supplementary Information 2.

## Data Availability

All sequences analyzed during this study has been registered in the DNA Data Bank of Japan (Accession Number: LC672676–LC677040). All datas generated during this study are included in this published article and its Supplementary Information files.
