# Supplementary material for: Differences in the microbiota of oral rinse, lesion, and normal site samples from patients with mucosal abnormalities on the tongue
Source: Sci Rep. 2022 Oct 7;12:16839. doi: 10.1038/s41598-022-21031-8 (PMC9546904; doi:10.1038/s41598-022-21031-8)
Supplement: Supplementary file 1 — Supplementary Information 1. [file 41598_2022_21031_MOESM1_ESM.docx]

**Supplementary Information**

**Differences in the microbiota of oral rinse, lesion, and normal site samples from patients with mucosal abnormalities on the tongue**

**Authors:** Yawaka Shitozawa^1, 2 *^, DDS, Kaoru Haro^1^, MD, PhD, Midori Ogawa^1^, PhD, Akihiko Miyawaki^2^, DDS, PhD, Mitsumasa Saito^1^, MD, PhD, Kazumasa Fukuda^1 *^, PhD

**Affiliations:** ^1^ Department of Microbiology, School of Medicine, University of Occupational and Environmental Health, Japan, 1-1 Iseigaoka, Yahatanishi-ku, Kitakyushu, Fukuoka, 807-8555, Japan, ^2^ Department of Dentistry and Oral Surgery, Hospital of the University of Occupational and Environmental Health, Japan, 1-1 Iseigaoka, Yahatanishi-ku, Kitakyushu, Fukuoka, 807-8556, Japan

**Corresponding author**

Correspondence to Yawaka Shitozawa & Kazumasa Fukuda.

Supplementary Tables S1-S5

Supplementary Figures S1-S15

**Supplementary Table S1.** Annotation of representative sequences of OTUs using the classifier on the RDPII.

| **OTU** | **Genus** |
| --- | --- |
| **OTU 0** | *Streptococcus* |
| **OTU 1** | *Gemella* |
| **OTU 2** | *Streptococcus* |
| **OTU 3** | *Neisseria* |
| **OTU 4** | *Streptococcus* |
| **OTU 5** | *Neisseria* |
| **OTU 6** | *Neisseria* |
| **OTU 7** | *Haemophilus* |
| **OTU 8** | *Fusobacterium* |
| **OTU 9** | *Streptococcus* |
| **OTU 10** | *Gemella* |
| **OTU 11** | *Granulicatella* |
| **OTU 12** | *Streptococcus* |
| **OTU 15** | *Cutibacterium.* |

**Supplementary Table S2.** Number and rate of sequence reads based on rarefied OTUs in three sample types.

| **OTU** | **Number of**  **sequence reads** | **Rate of**  **sequence reads (%)** |
| --- | --- | --- |
| **OTU 0** | 1047 | 30.88 |
| **OTU 1** | 203 | 5.99 |
| **OTU 2** | 121 | 3.57 |
| **OTU 3** | 88 | 2.60 |
| **OTU 4** | 67 | 1.98 |
| **OTU 5** | 54 | 1.59 |
| **OTU 8** | 51 | 1.50 |
| **OTU 9** | 50 | 1.47 |
| **OTU 6** | 48 | 1.42 |
| **OTU 7** | 45 | 1.33 |
| **OTU 12** | 40 | 1.18 |
| **OTU 11** | 39 | 1.15 |
| **OTU 10** | 36 | 1.06 |
| **others** | 1501 | 44.28 |
| **Total** | 3390 | 100.00 |

**Supplementary Table S3.** Number and rate of sequence reads based on rarefied OTUs in two swab samples.

| **OTU** | **Number of**  **sequence reads** | **Rate of**  **sequence reads (%)** |
| --- | --- | --- |
| **OTU 0** | 860 | 38.05 |
| **OTU 1** | 190 | 8.41 |
| **OTU 2** | 66 | 2.92 |
| **OTU 3** | 51 | 2.26 |
| **OTU 6** | 48 | 2.12 |
| **OTU 7** | 40 | 1.77 |
| **OTU 4** | 37 | 1.64 |
| **OTU 8** | 37 | 1.64 |
| **OTU 9** | 34 | 1.50 |
| **OTU 12** | 27 | 1.19 |
| **OTU 5** | 26 | 1.15 |
| **OTU 15** | 25 | 1.11 |
| **others** | 819 | 36.24 |
| **Total** | 2260 | 100.00 |

**Supplementary Table S4.** Number and rate of sequence reads of genus level in three sample types.

| **Genus** | **Number of**  **sequence reads** | **Rate of**  **sequence reads (%)** |
| --- | --- | --- |
| ***Streptococcus*** | 1950 | 57.52 |
| ***Gemella*** | 297 | 8.76 |
| ***Neisseria*** | 216 | 6.37 |
| ***Fusobacterium*** | 101 | 2.98 |
| ***Prevotella*** | 83 | 2.45 |
| ***Haemophilus*** | 78 | 2.30 |
| ***Neisseriaceae* unclassified bacterium** | 66 | 1.95 |
| ***Veillonella*** | 64 | 1.89 |
| ***Granulicatella*** | 63 | 1.86 |
| ***Propionibacterium*** | 42 | 1.24 |
| ***Leptotrichia*** | 37 | 1.09 |
| ***Sphingomonas*** | 36 | 1.06 |
| **others** | 357 | 10.53 |
| **Total** | 3390 | 100.00 |

**Supplementary Table S5.** Number and rate of sequence reads of genus level in two swab samples.

| **Genus** | **Number of**  **sequence reads** | **Rate of**  **sequence reads (%)** |
| --- | --- | --- |
| ***Streptococcus*** | 1295 | 57.30 |
| ***Gemella*** | 245 | 10.84 |
| ***Neisseria*** | 105 | 4.65 |
| ***Fusobacterium*** | 69 | 3.05 |
| ***Haemophilus*** | 65 | 2.88 |
| ***Neisseriaceae* unclassified bacterium** | 59 | 2.61 |
| ***Propionibacterium*** | 42 | 1.86 |
| ***Veillonella*** | 38 | 1.68 |
| ***Sphingomonas*** | 36 | 1.59 |
| ***Prevotella*** | 35 | 1.55 |
| ***Granulicatella*** | 31 | 1.37 |
| ***Enhydrobacter*** | 28 | 1.24 |
| ***Leptotrichia*** | 25 | 1.11 |
| **others** | 187 | 8.27 |
| **Total** | 2260 | 100.00 |


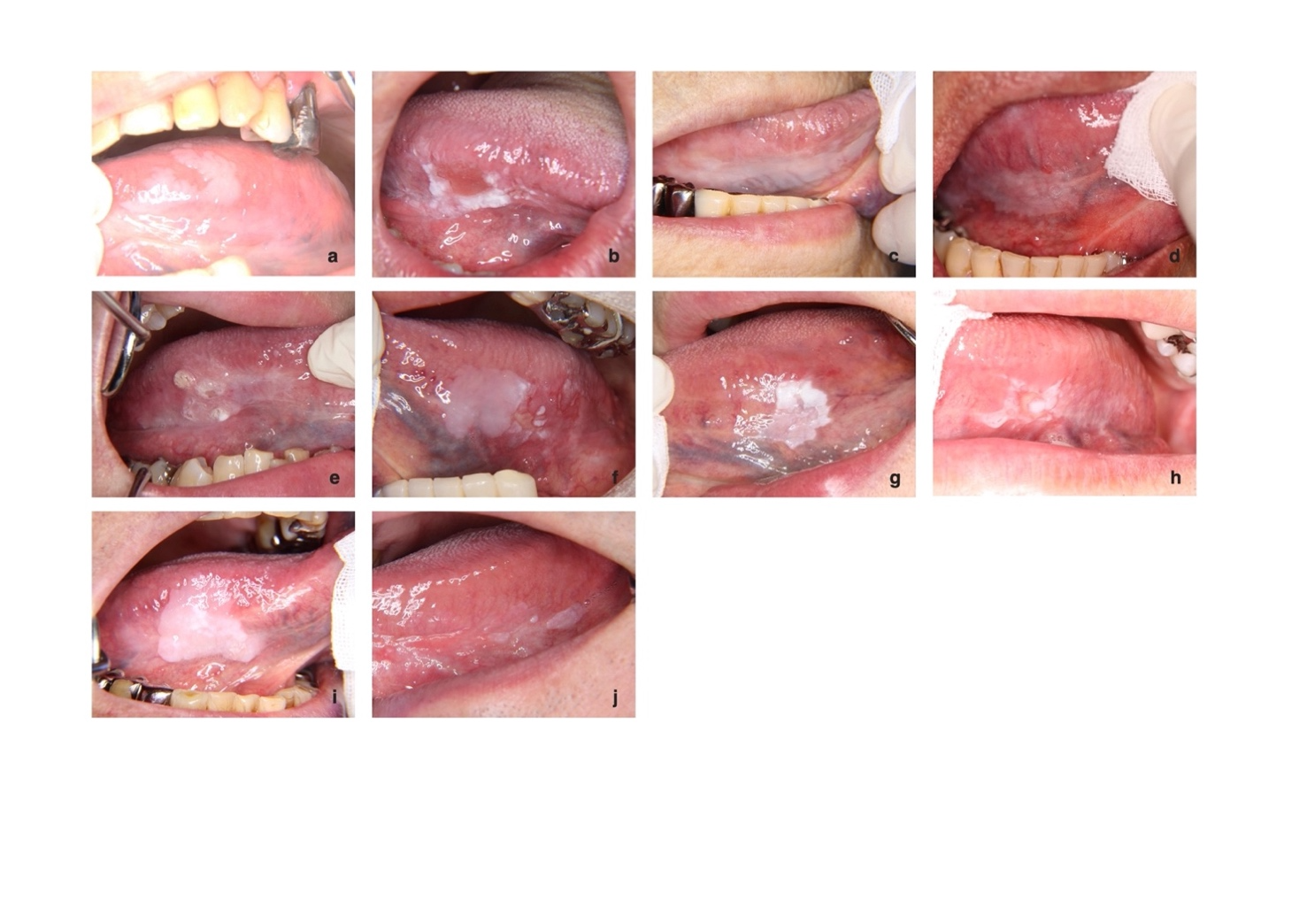


**Supplementary Figure S1.** Graphical characteristics of lesions on the tongue.

(a) Patient 1, (b) Patient 2, (c) Patient 3, (d) Patient 4, (e) Patient 5, (f) Patient 6, (g) Patient 7, (h) Patient 8, (i) Patient 9, and (j) Patient 10.


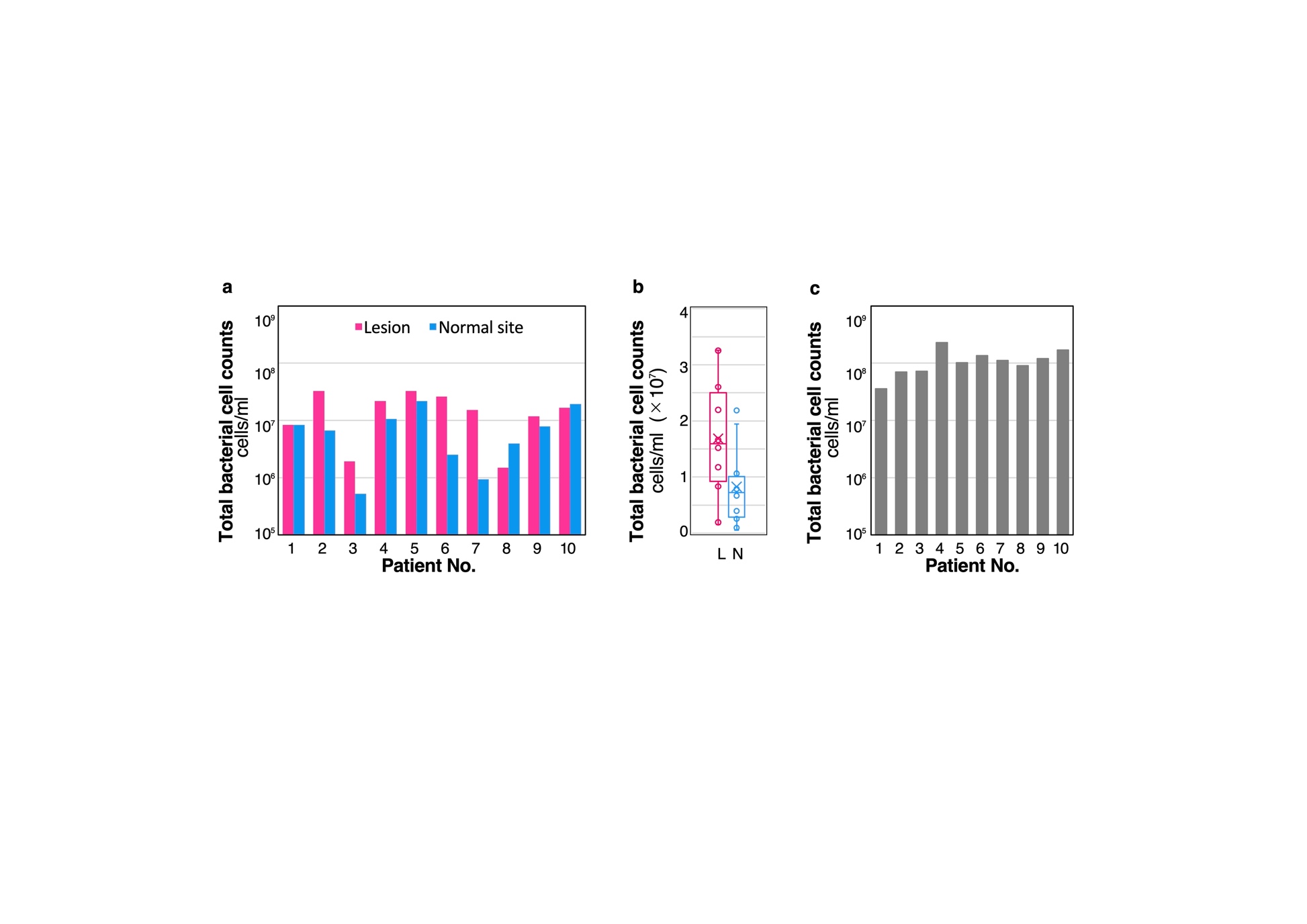


**Supplementary Figure S2.** Total bacterial cell counts in each sample.

(a) Bacterial cell counts in lesion and normal site swabs. (b) Comparison of bacterial cell counts in lesion and normal site swabs. The box plots show the 25th and 75th quartiles (bottom and top of the box, respectively), median (middle horizontal line), average (cross mark), and minimum and maximum values that are not outliers (top and bottom whiskers). Outliers are defined as 1.5 times the quartile range (points). Statistical significance between two type samples was tested using Mann-Whitney's U test. p < 0.05. L: lesion, N: normal site. (c) Bacterial cell counts in oral rinse samples.


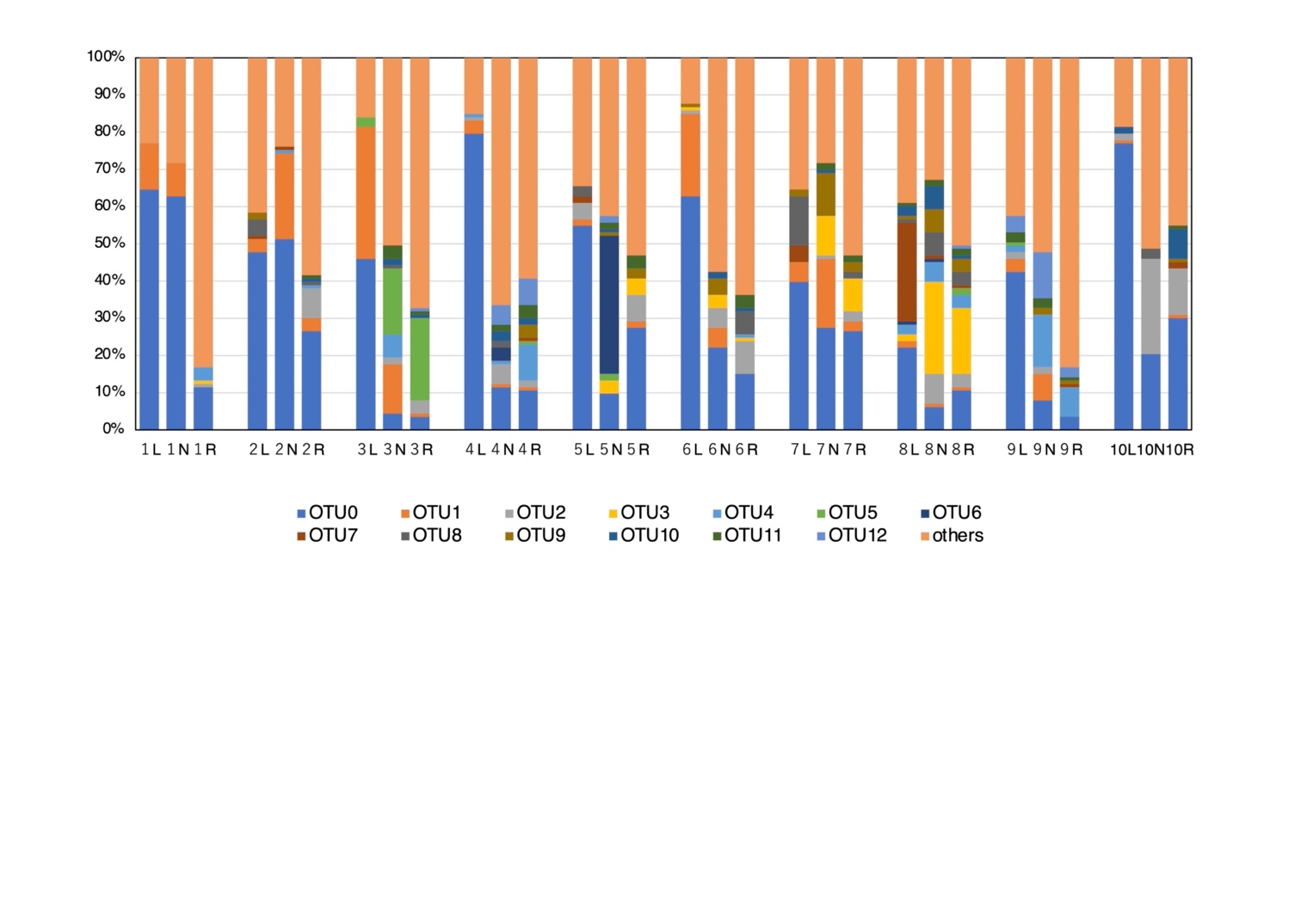


**Supplementary Figure S3.** Relative abundance based on OTUs level in each sample.

Graph showing the abundance of 13 OTUs and Others (OTUs with relative abundance < 1% in the total sequences). All oral rinse samples had high percentage of Others. Data labels show number of patient. L: lesion, N: normal site, R: oral rinse, OTU: operational taxonomic unit.


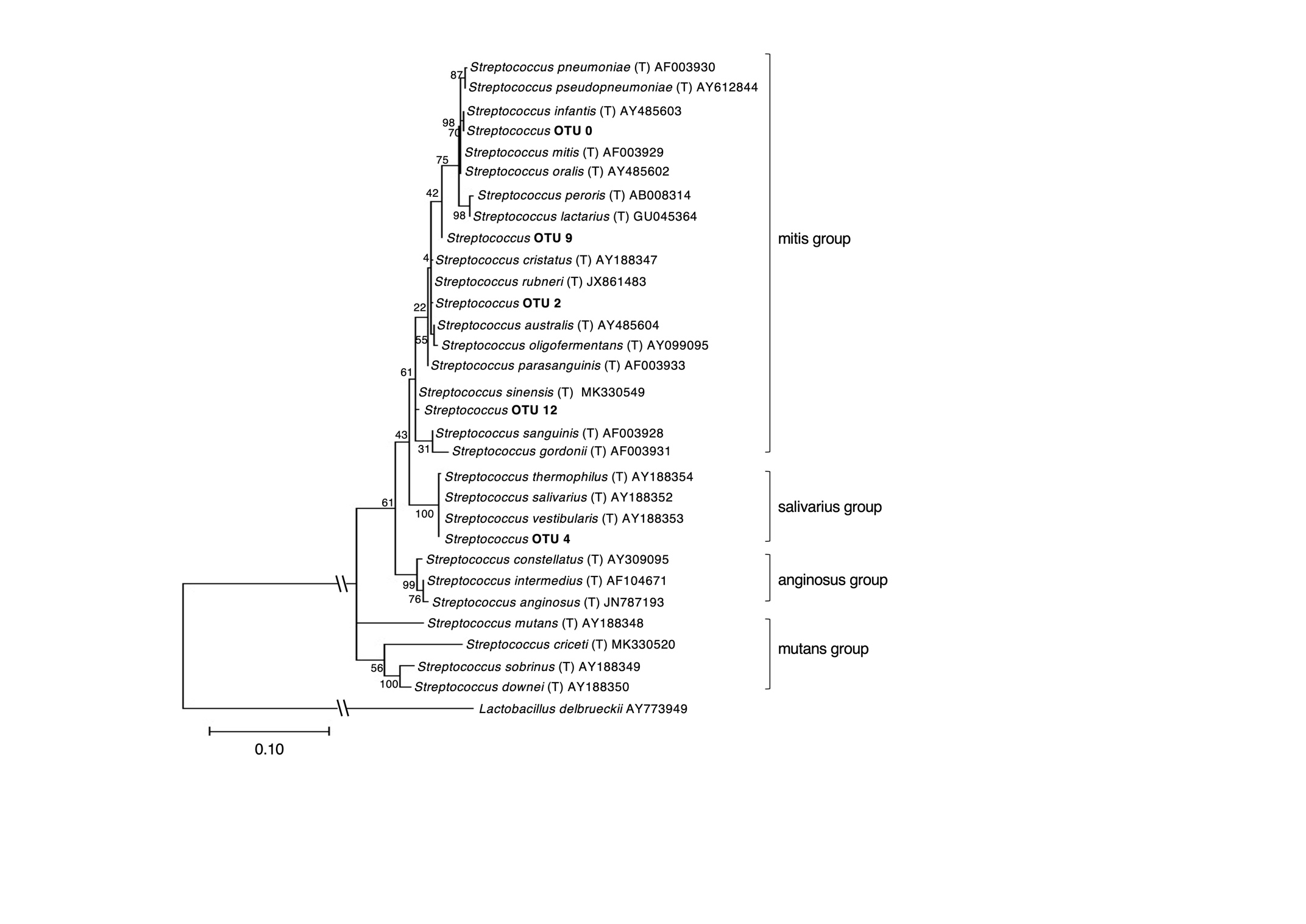


**Supplementary Figure S4.** Phylogenetic analysis of OTU 0,2,4,9,12 assigned as the genus *Streptococcus.*

The 16S rRNA gene sequences assigned to the genus *Streptococcus* using the classifier on the RDPII were aligned with those of the type strains of the genus *Streptococcus* using MEGA-X. A maximum likelihood tree based on the alignments (550 bp) was constructed using MUSCLE. The K2+G+I model (K2: Kimura 2-parameter, G+I: Gamma distribution with invariant sites) selected using the Find best model was used. Accession numbers are provided according to the taxonomic name. OTUs obtained in this study are shown in bold font. *Lactobacillus delbrueckii* AY773949 was used as an outgroup. Scale bar denotes substitutions per site. Bootstrap values from 500 analyses are shown at the branch points. OTU: operational taxonomic unit.


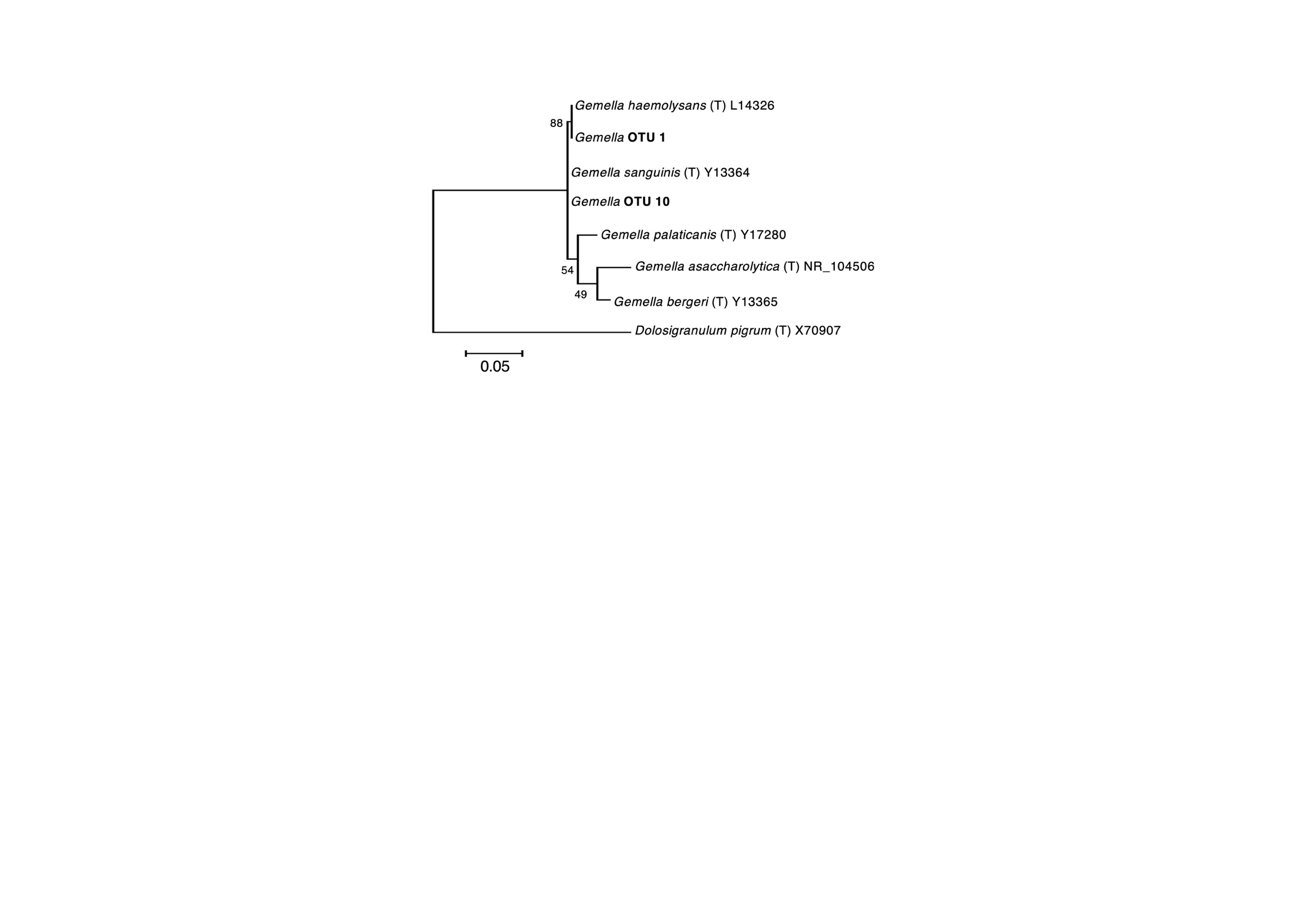


**Supplementary Figure S5.** Phylogenetic analysis of OTU 1,10 assigned as the genus *Gemella.*

The 16S rRNA gene sequences assigned to the genus *Gemella* using the classifier on the RDPII were aligned with those of type strains of the genus *Gemella* using MEGA-X. A maximum likelihood tree based on the alignments (548 bp) was constructed using MUSCLE. The K2+G (K2: Kimura 2-parameter, G: Gamma distribution) selected using the Find best model was used. Accession numbers are provided according to the taxonomic name. OTUs obtained in this study are shown in bold font. *Dolosigranulum pigrum* (T) X70907 was used as an outgroup. Scale bar denotes substitutions per site. Bootstrap values from 500 analyses are shown at the branch points. OTU: operational taxonomic unit.


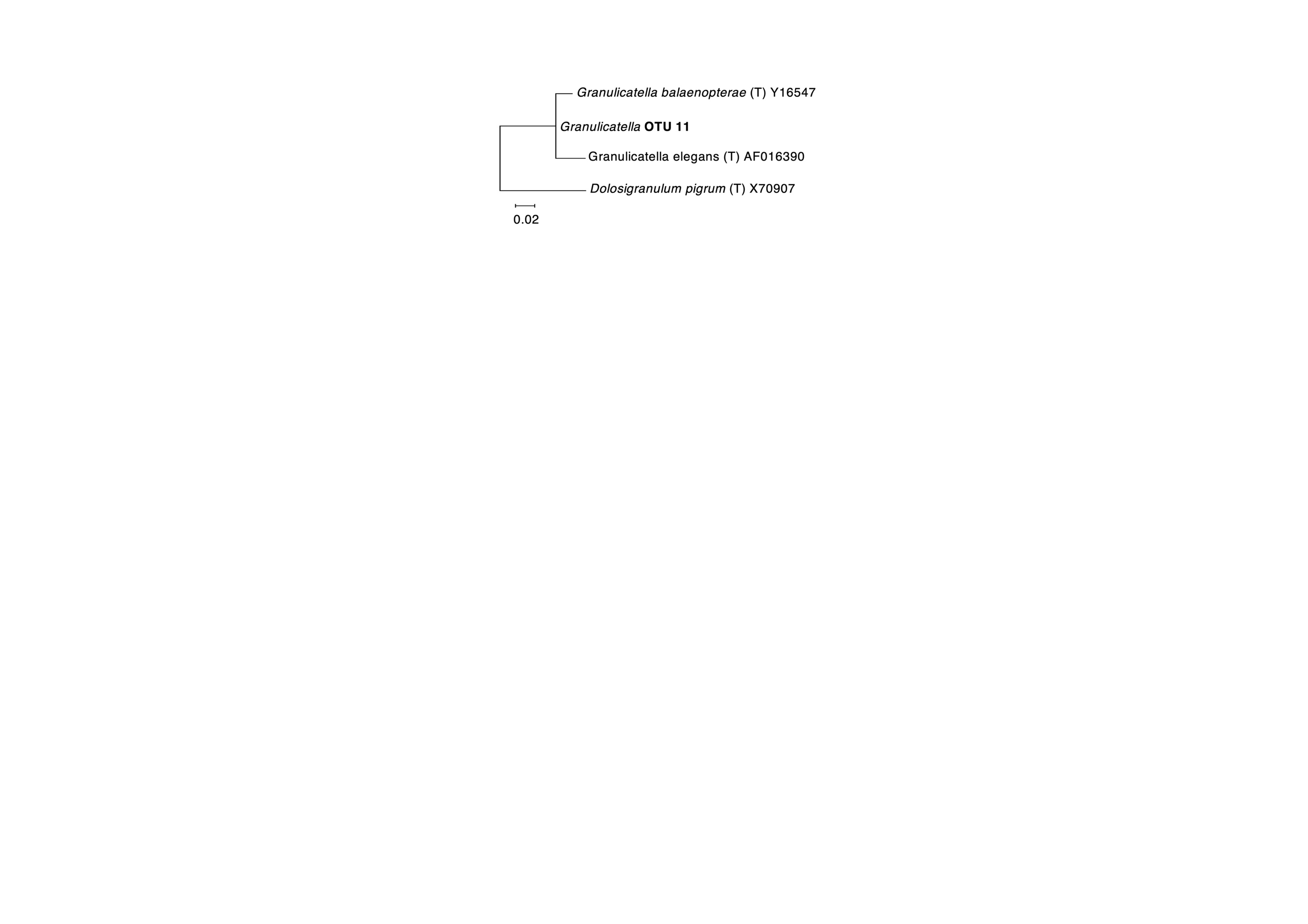


**Supplementary Figure S6.** Phylogenetic analysis of OTU 11 assigned as the genus *Granulicatella.*

The 16S rRNA gene sequence assigned to the genus *Granulicatella* using the classifier on the RDPII was aligned with those of type strains of the genus *Granulicatella* using MEGA-X. A maximum likelihood tree based on the alignments (550 bp) was constructed using MUSCLE. The K2+G model (K2: Kimura 2-parameter, G: Gamma distribution) selected using the Find best model was used. Accession numbers are provided according to the taxonomic name. OTUs obtained in this study are shown in bold font. *Dolosigranulum pigrum* (T) X70907 was used as an outgroup. Scale bar denotes substitutions per site. Bootstrap values from 500 analyses are shown at the branch points. OTU: operational taxonomic unit.


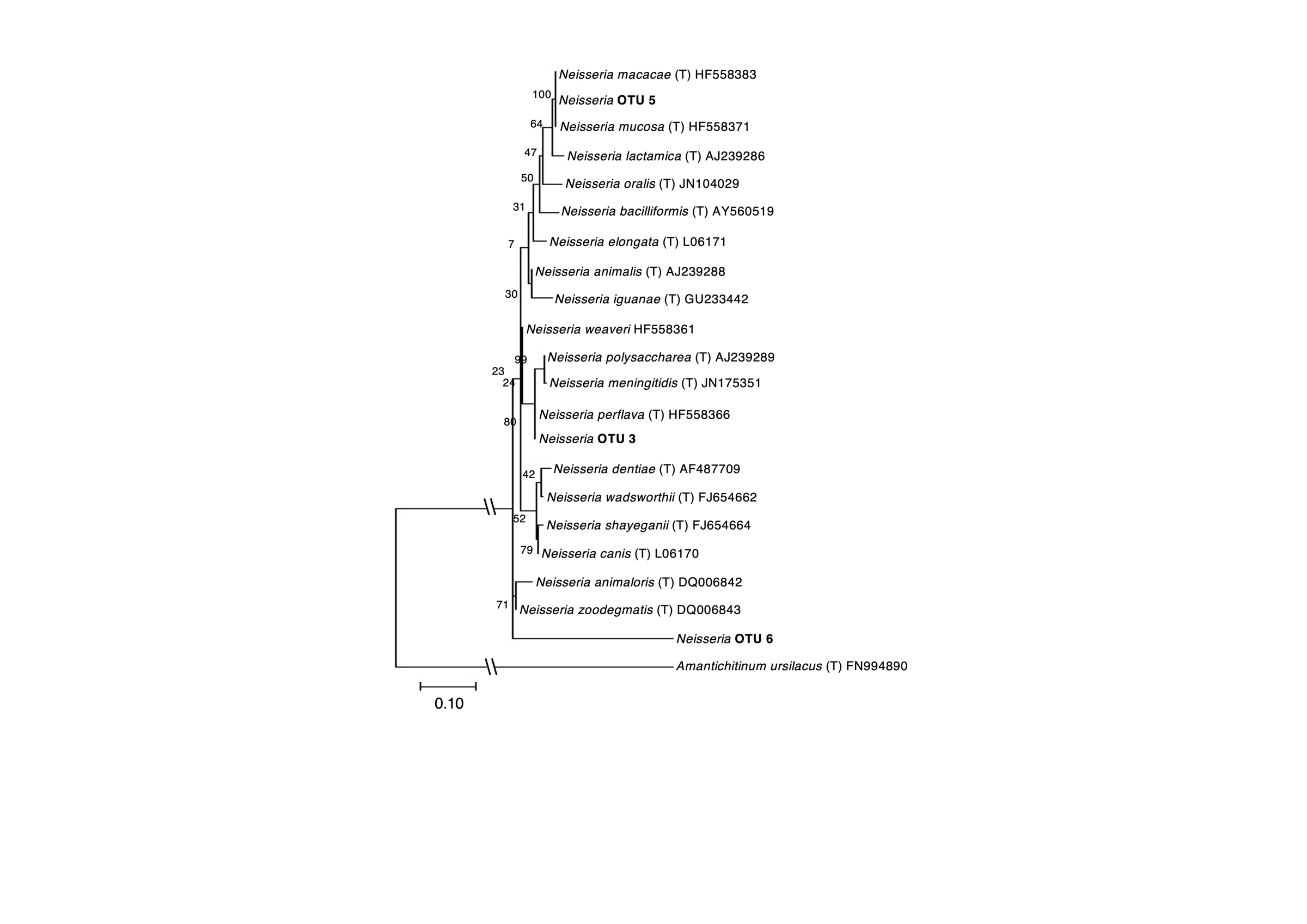


**Supplementary Figure S7.** Phylogenetic analysis of the OTU 3,5,6 assigned as the genus *Neisseria.*

The 16S rRNA gene sequences assigned to *the* genus *Neisseria* using the classifier on the RDPII were aligned with those of type strains of the genus *Neisseria* using MEGA-X. A maximum likelihood tree based on the alignments (550 bp) was constructed using MUSCLE. The K2+G+I model (K2: Kimura 2-parameter, G+I: Gamma distribution with invariant sites) selected using the Find best model was used. Accession numbers are provided according to the taxonomic name. OTUs obtained in this study are shown in bold font. *Amantichitinum ursilacus* (T) FN994890 was used as an outgroup. Scale bar denotes substitutions per site. Bootstrap values from 500 analyses are shown at the branch points. OTU: operational taxonomic unit.


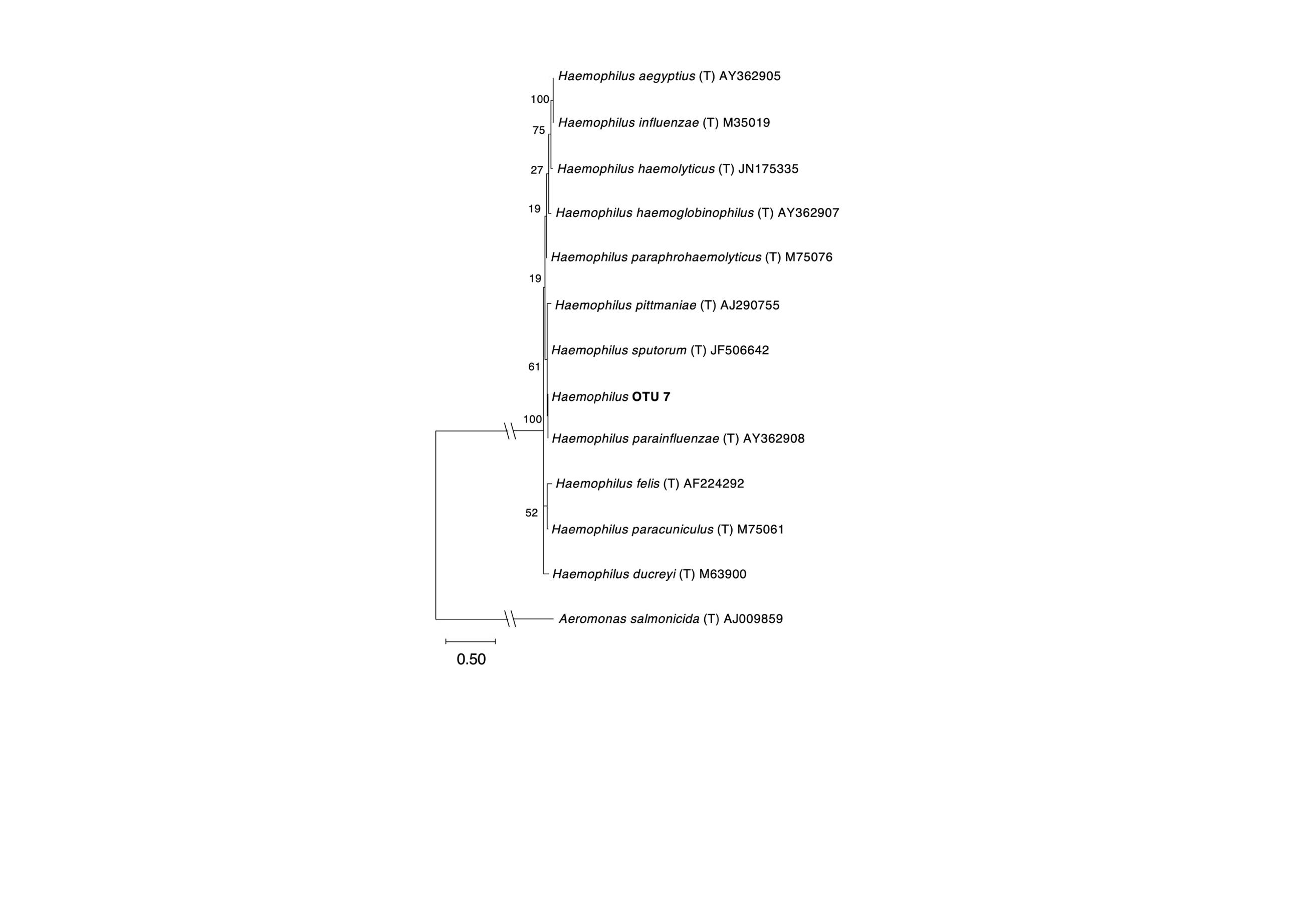


**Supplementary Figure S8.** Phylogenetic analysis of OTU 7 assigned as the genus *Haemophilus.*

The 16S rRNA gene sequence assigned to the genus *Haemophilus* using the classifier on the RDPII was aligned with those of type strains of the genus *Haemophilus* using MEGA-X. A maximum likelihood tree based on the alignments (547 bp) was constructed using MUSCLE. The K2+G+I model (K2: Kimura 2-parameter, G+I: Gamma distribution with invariant sites) selected using the Find best model was used. Accession numbers are provided according to the taxonomic name. OTUs obtained in this study are shown in bold font. *Aeromonas salmonicida* (T) AJ009859 was used as an outgroup. Scale bar denotes substitutions per site. Bootstrap values from 500 analyses are shown at the branch points. OTU: operational taxonomic unit.


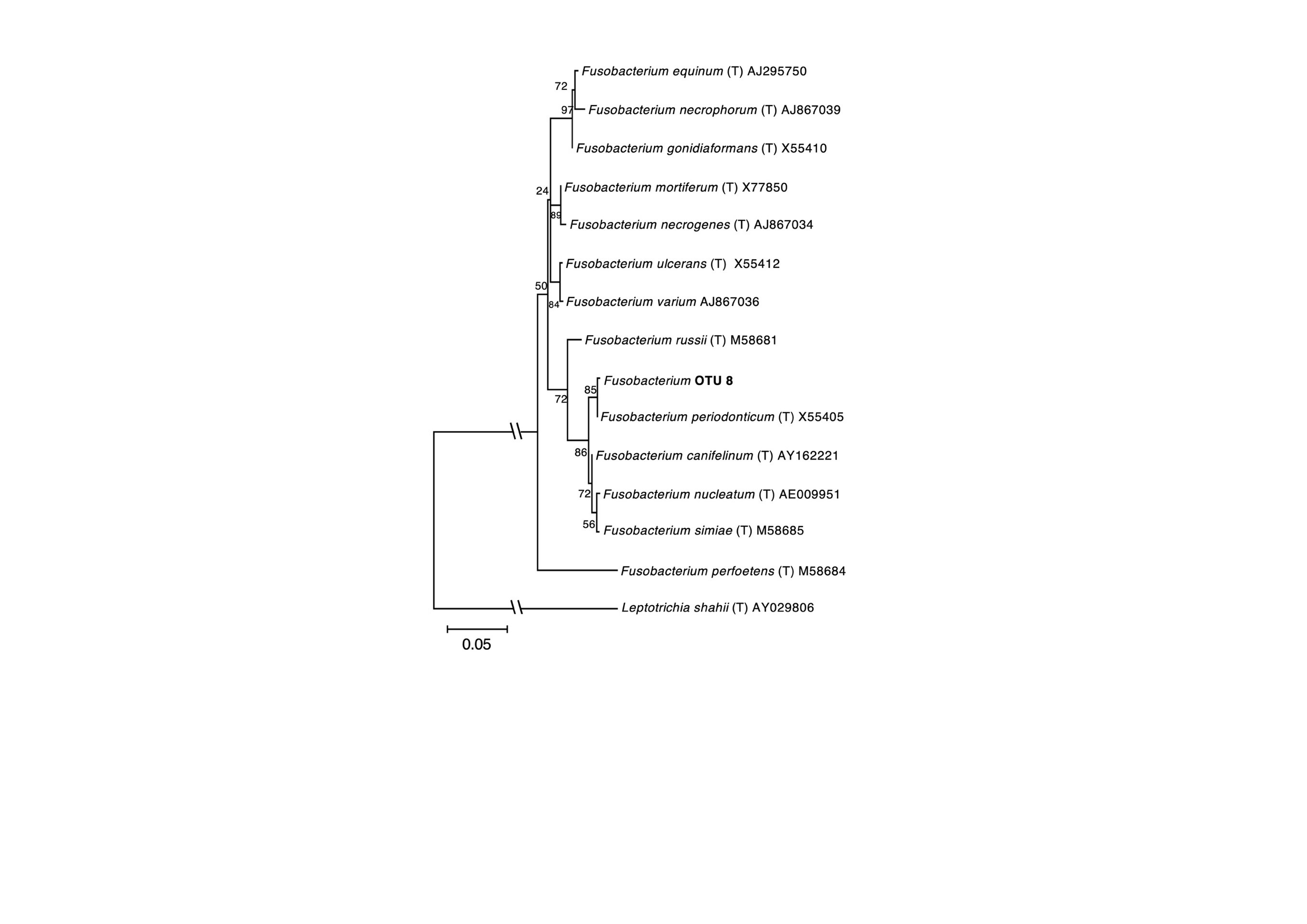


**Supplementary Figure S9.** Phylogenetic analysis of OTU 8 assigned as the genus *Fusobacterium.*

The 16S rRNA gene sequence assigned to the genus *Fusobacterium* using the classifier on the RDPII in this study was aligned with those of the type strains of the genus *Fusobacterium* using MEGA-X. A maximum likelihood tree based on the alignments (524 bp) was constructed using MUSCLE. The K2+G model (K2: Kimura 2-parameter, G: Gamma distribution) selected using the Find best model was used. Accession numbers are provided according to the taxonomic name. OTUs obtained in this study are shown in bold font. *Leptotrichia shahii* (T) AY029806 was used as the outgroup. Scale bar denotes substitutions per site. Bootstrap values from 500 analyses are shown at the branch points. OTU: operational taxonomic unit.


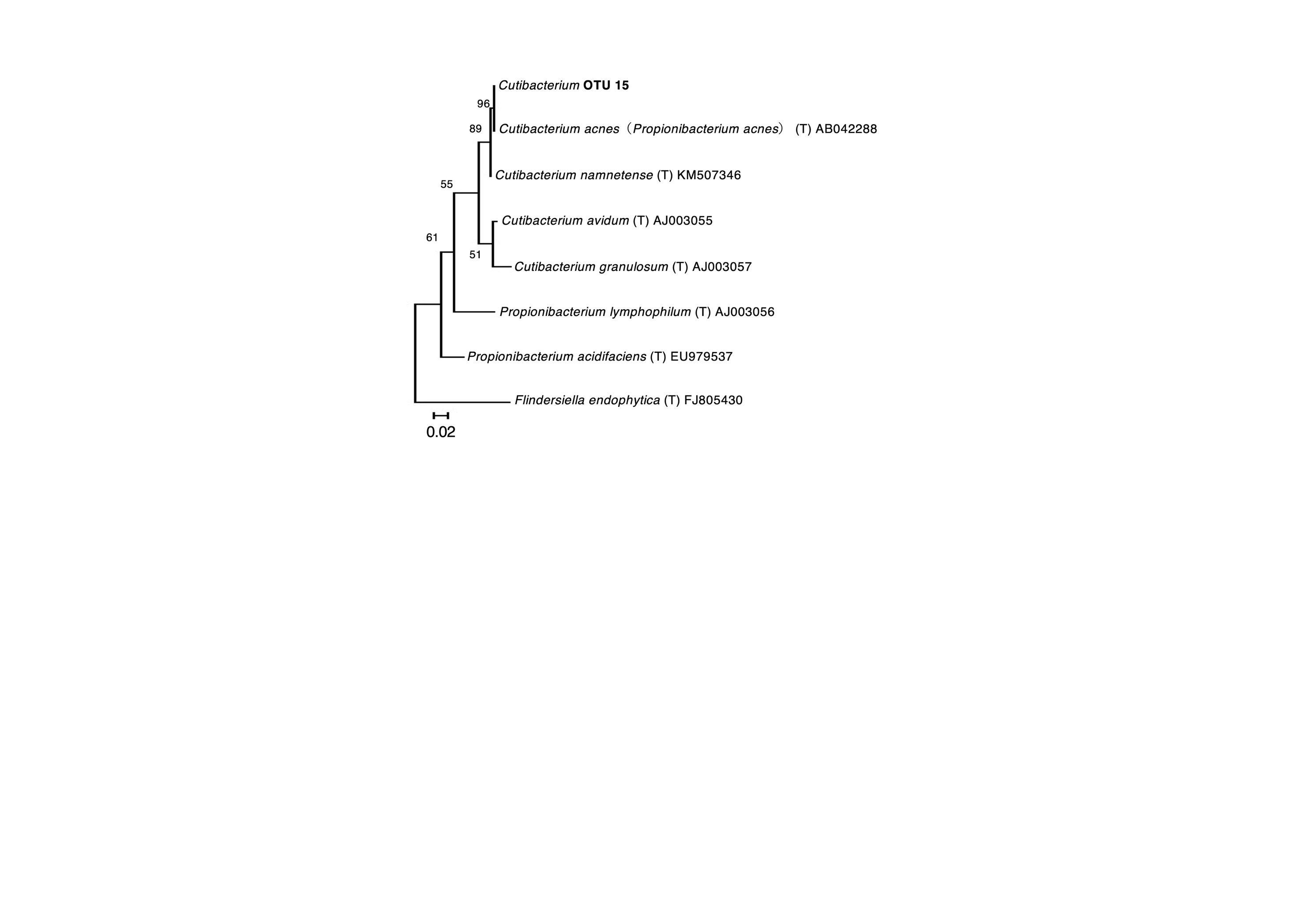


**Supplementary Figure S10.** Phylogenetic analysis of OTU 15 assigned as the genus *Cutibacterium*.

The 16S rRNA gene sequence assigned to the genus *Cutibacterium* using the classifier on the RDPII was aligned with those of type strains of the genus *Cutibacterium* and *Propionibacterium* using MEGA-X. A maximum likelihood tree based on the alignments (531 bp) was constructed using MUSCLE. The HKY+G model (HKY: Hasegawa-Kishino-Yano, G: Gamma distribution) selected using the Find best model was used. Accession numbers are provided according to the taxonomic name. OTUs obtained in this study are shown in bold font. *Flindersiella endophytica* FJ805430 was used as the outgroup. Scale bar denotes substitutions per site. Bootstrap values from 500 analyses are shown at the branch points. OTU: operational taxonomic unit.

**
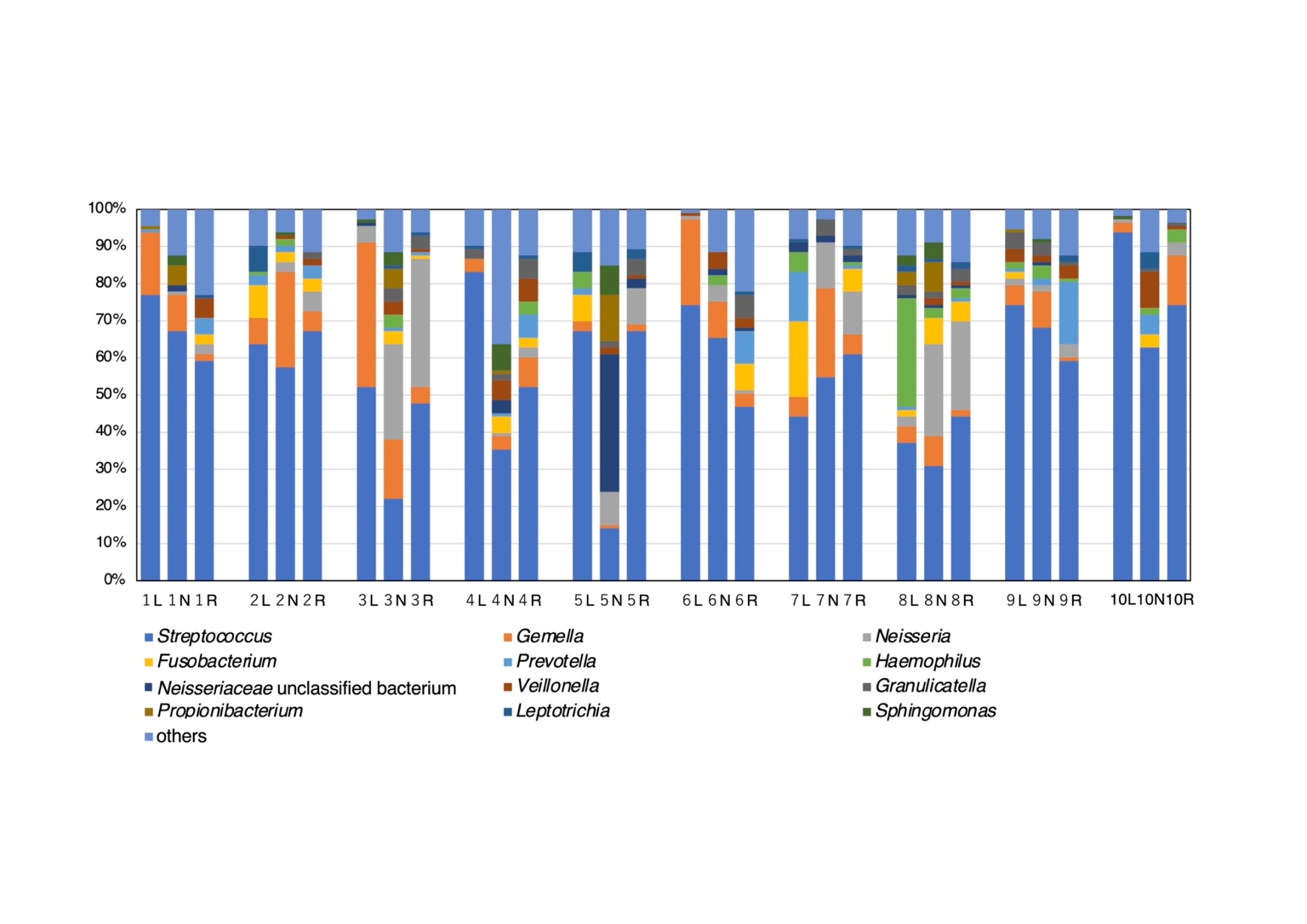
**

**Supplementary Figure S11.** Relative abundance at genus level in each sample.

Graph showing the abundance of 11 genera and *Neisseriaceae* unclassified bacterium and others (genus with relative abundance < 1.0% in the total sequences). The genus *Streptococcus* was the most abundant in most samples. Data labels show the patient number. L: lesion, N: normal site, R: oral rinse.


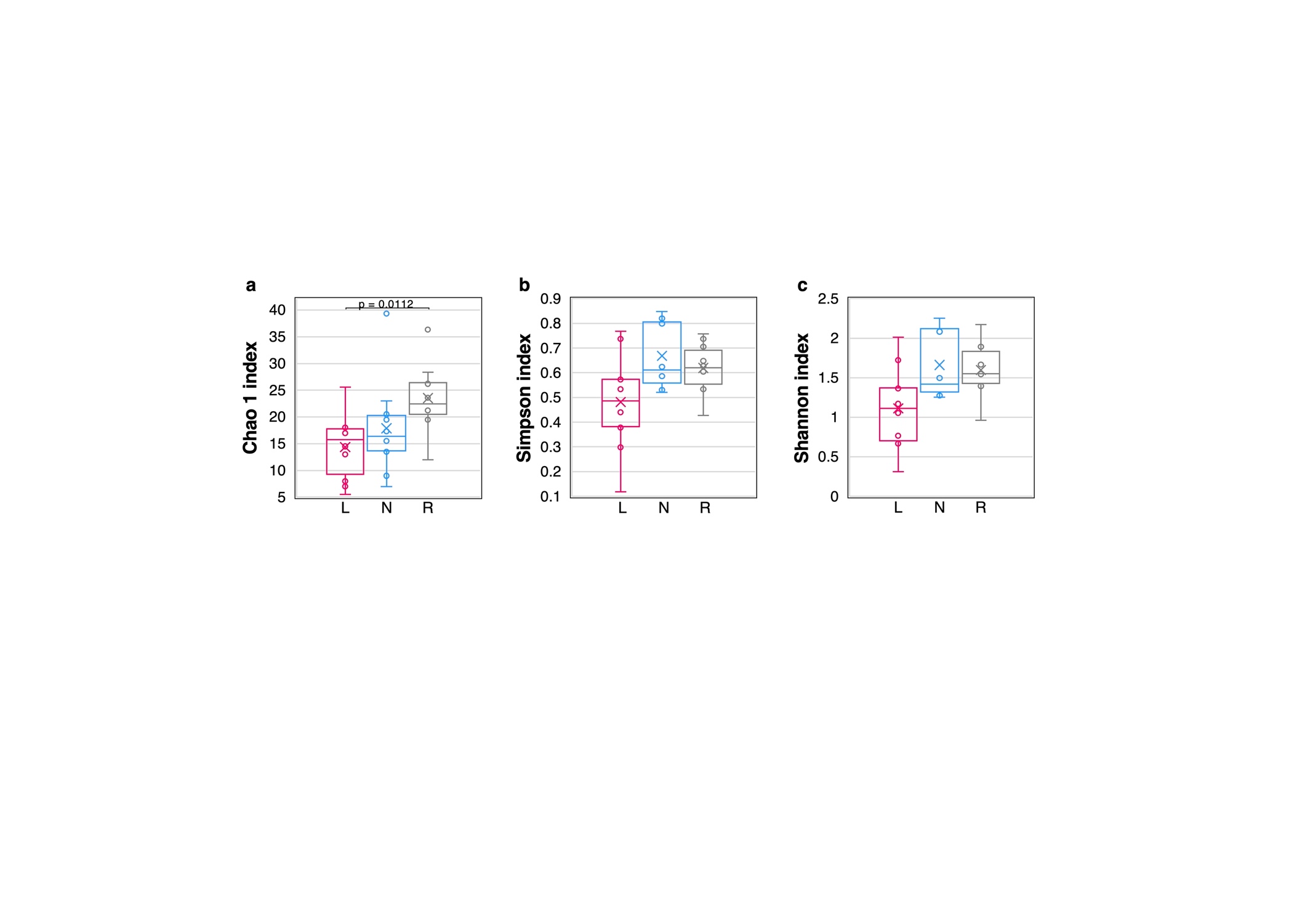


**Supplementary Figure S12.** Comparison of alpha diversity based on genus level.

(a) Chao1 index, (b) Simpson index, and (c) Shannon index. The box plots show the 25th and 75th percentiles (bottom and top of the box, respectively), median (middle horizontal line), average (cross mark), and minimum and maximum values that are not outliers (top and bottom whiskers). Outliers are defined as values greater than 1.5 × the interquartile range (points). Statistical significance among the three types of samples was tested using the Kruskal-Wallis test. For multiple comparisons, the Steel-Dwass test was applied (bars, p < 0.05). L: lesion, N: normal site, R: oral rinse.


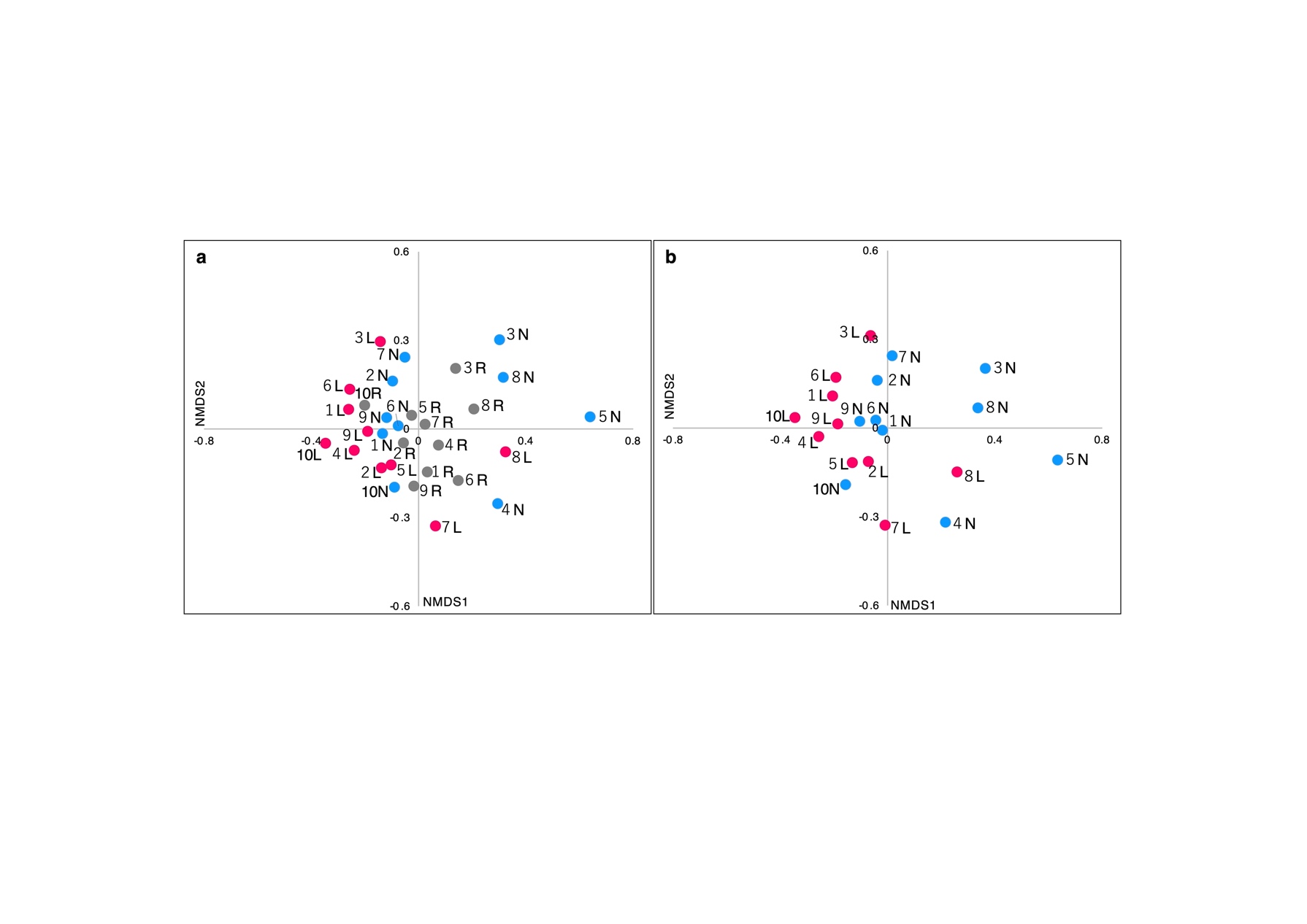


**Supplementary Figure S13.** Nonmetric multidimensional scaling plots based on genus level.

(a) Three sample types. (b) Two swab samples. Nonmetric multidimensional scaling plots were generated from a distance matrix of Bray-Curtis dissimilarity values based on genus level. Each point corresponds to a single sample. Lesion samples are plotted as pink dots, normal site samples as blue dots, and oral rinse samples as gray dots. Data labels show the patient number. L: lesion, N: normal site, R: oral rinse.

**
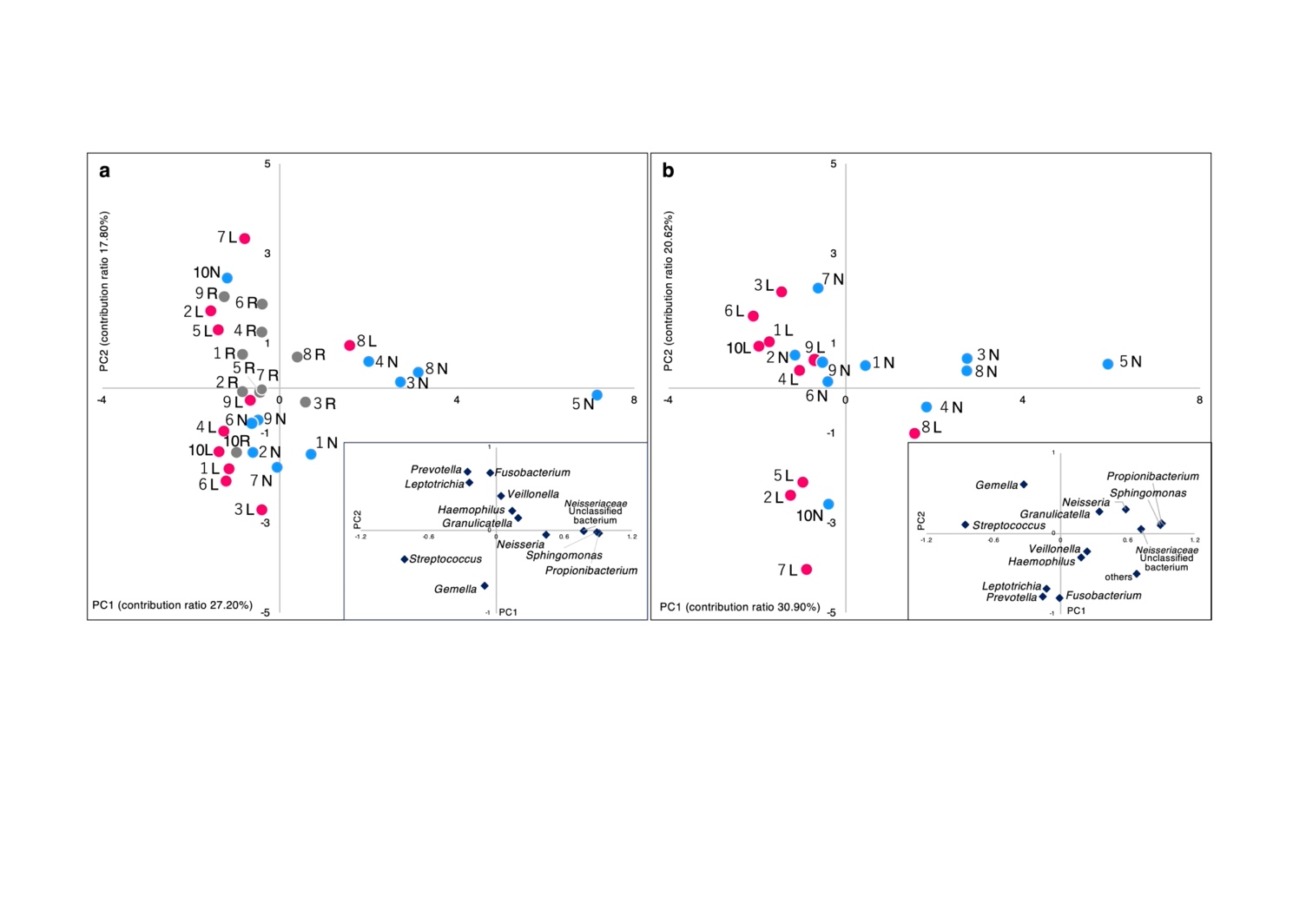
**

**Supplementary Figure S14.** Principal component analysis based on genus level.

(a) Three sample types. (b) Two swab samples. The horizontal axis shows PC1, and the vertical axis shows PC2. The eigenvector is shown in the box below. Lesions are plotted as pink dots, normal sites as blue dots, and oral rinse samples as grey dots. Data labels show the patient number. L: lesion, N: normal site, R: oral rinse, PC1: first principal component, PC2: second principal component.


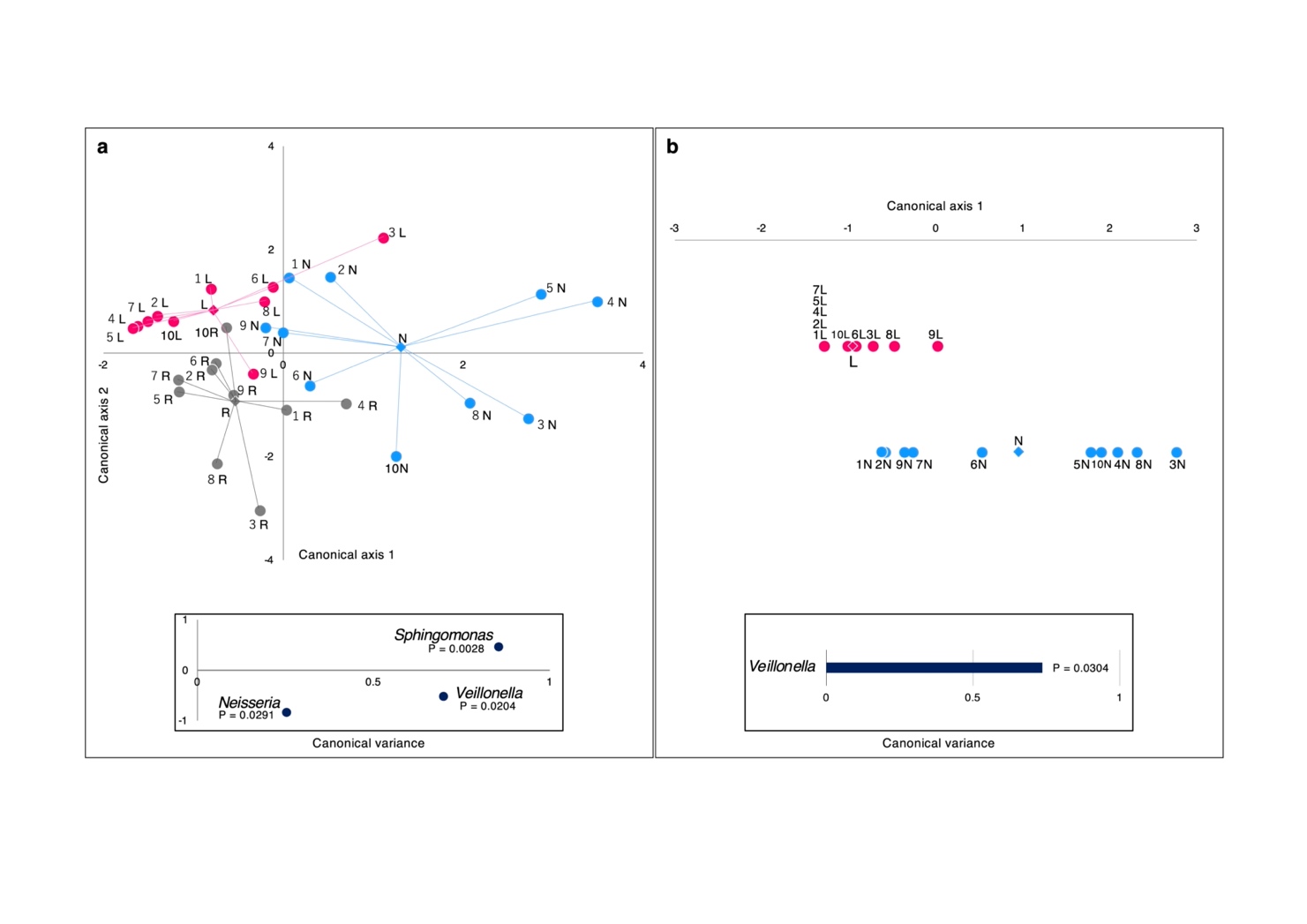


**Supplementary Figure S15.** Canonical discriminant analysis based on genus level.

(a) Three sample types. (b) Two swab samples. Each point represents the canonical score (CS) of each sample based on the respective canonical variables. The X-axis is canonical axis 1, and the Y-axis is canonical axis 2. Lesions are plotted as pink dots, normal sites as blue dots, oral rinse samples as gray dots, and each center of gravity as diamonds. Data labels show the patient number. L: lesion, N: normal site, R: oral rinse. Canonical discriminant function, p < 0.05.
